# Characterizing Cancer Symptom Experiences and Related Psychosocial Concerns Through Chinese Social Media Narratives: Computational Text Analysis

**DOI:** 10.2196/102862

**Published:** 2026-10-20

**Authors:** Huan Yu, Yin Wang, Jiaqi Ding, Ying Wang, Yi Zhang, Yaxi Wang, Jingfang Hong

**Affiliations:** 1 Department of Epidemiology and Biostatistics, School of Public Health, Anhui Medical University, Hefei, Anhui, China; 2 School of Nursing, Anhui Medical University, No. 26 Xinglin Road, Feixi County, Hefei, Anhui, 231200, China, 86 055165161009; 3 Nursing International Collaboration Research Center of Anhui Province, Hefei, Anhui, China

**Keywords:** patient-reported outcomes, patient-generated health data, cancer symptoms, social media, oncology nursing, topic modeling, supportive care

## Abstract

**Background:**

Patients with cancer frequently experience complex and multidimensional symptom burdens. Standardized patient-reported outcome measures provide structured approaches to symptom assessment but may not capture the full range of symptom-related and psychosocial concerns expressed in patients’ narratives. Patient-generated social media data offer a complementary source for examining how these concerns are expressed online.

**Objective:**

This study aimed to characterize cancer symptom–related narratives in Chinese social media posts using a conventional symptom assessment framework and to identify additional experiential themes through neural topic modeling.

**Methods:**

A computational text analysis integrating large language model (LLM) annotation and neural topic modeling was conducted. A corpus of 26,086 first-person patient narratives was collected from Xiaohongshu and Zhihu between November 2013 and February 2026 using cancer-related search terms. First-person narratives and 6 SPPADE (sleep disturbance, pain, physical function impairment, anxiety, depression, and low energy or fatigue) symptom domains were identified using GPT-4o-mini (OpenAI) and validated against independent human coding. Symptom occurrence, co-occurrence, multiplicity, and coping strategies were characterized, with exploratory stratified analyses by cancer type. BERTopic modeling was applied to identify latent themes, platform sensitivity analyses, and temporal distribution were examined.

**Results:**

For first-person narrative identification, human raters showed 89.2% agreement (Cohen κ=0.784); the LLM achieved an *F*_1_-score of 81.2% against the adjudicated reference standard. For SPPADE extraction, mean Cohen κ values for LLM-human agreement were 0.714 (SD 0.114) and 0.754 (SD 0.095) across the 6 symptom domains. Overall, 13,896 of 26,086 (53.3%) posts contained at least 1 identifiable symptom. Pain was the most frequently identified symptom (5783/26,086, 22.2%), followed by physical function impairment (4153/26,086, 15.9%) and depression (4050/26,086, 15.5%). Pain and physical function impairment formed the most frequent co-occurring symptom pair (n=1407). Coping strategies were described in 7104/26,086 (27.2%) posts; medication-based approaches were most prominent, particularly for pain and sleep disturbance, while social and psychological support were more common for anxiety and depression. BERTopic modeling identified 13 latent themes, including cancer treatment trajectories, health care system interaction, existential distress, family involvement, and financial burden, highlighting experiential concerns that may be underrepresented in conventional symptom assessment. The proportion of posts containing at least 1 SPPADE symptom was similar between Xiaohongshu and Zhihu (53.2% vs 54.1%; *P*=.35), although individual symptom distributions differed across platforms.

**Conclusions:**

Chinese social media posts contained multidimensional symptom-related narratives alongside psychosocial and experiential concerns that may be less visible in structured symptom assessment. Patient-generated social media data may therefore provide a complementary source for understanding cancer symptom experiences and supportive care concerns. However, differences in platform composition, uneven temporal coverage, and the nonrepresentativeness of social media users limit generalizability, and the clinical utility of integrating such data into routine symptom assessment requires further evaluation.

## Introduction

Cancer remains a major global public health challenge, with approximately 20 million new cases and 9.7 million deaths reported worldwide in 2022, and incidence projected to rise substantially by 2050 as populations age and exposure to risk factors intensifies [1-3]. Beyond its epidemiological scale, cancer imposes substantial and multidimensional symptom burdens on patients across the entire illness trajectory, from diagnosis through active treatment and into long-term survivorship [4,5]. Symptom burden encompasses both the severity of individual symptoms and the cumulative impact on daily functioning and well-being. Physical symptoms, including pain, fatigue, and functional impairment, frequently co-occur with psychological and behavioral symptoms such as depression, anxiety, and sleep disturbance, generating a cumulative burden that exceeds the impact of individual symptoms. This burden is associated with reduced adherence to therapeutic interventions [6], diminished quality of life [7], and poorer clinical outcomes [8]. Symptoms are also dynamic, varying across treatment phases, cancer types, and disease stages, and are further shaped by individual, social, and health system factors [9-11].

Standardized patient-reported outcome measures (PROMs) have played a central role in advancing oncology symptom science and enabling scalable symptom assessment in clinical practice. Their integration into routine care has improved symptom detection and supported clinical decision-making. One prominent framework is SPPADE (sleep disturbance, pain, physical function impairment, anxiety, depression, and low energy or fatigue) symptom hexad, providing a clinically grounded structure for conceptualizing key domains of cancer symptoms [12,13]. Evidence from large-scale clinical implementation supports the value of systematic symptom monitoring. The E2C2 pragmatic trial, involving 24,874 patients across a multistate health system, found that systematic SPPADE monitoring supported by electronic health records and collaborative care was associated with reduced symptom burden and fewer emergency department visits and hospital admissions [12,14]. While PROMs helped patients articulate symptoms, they also raised questions about whether responses were reviewed and how they were interpreted by clinicians [15]. Clinicians tend to underestimate the symptom severity and fail to record or capture half of patients’ symptomatic adverse events [16], even within structured assessment contexts [17]. These findings suggest that structured assessment alone may not fully capture the range of symptom-related and psychosocial concerns expressed by patients [18].

Social media platforms have emerged as a valuable source of patient-generated health data, offering large-scale and relatively naturalistic accounts of illness experiences [19,20]. Platforms such as Xiaohongshu and Zhihu provide access to distinct but complementary forms of patient discourse. Xiaohongshu is characterized by personal experiential narratives, while Zhihu hosts more extended and deliberative discussions related to treatment decisions and health care navigation [21]. They may also provide access to concerns that are not routinely expressed or documented during clinical consultations, particularly where sociocultural norms discourage open disclosure. In China, where approximately 4.82 million new cancer cases were diagnosed in 2022, understanding patients’ symptoms, immediate concerns, and needs beyond clinical settings is particularly important given the scale and diversity of cancer care needs [19,22]. Cultural norms, including traditional health beliefs and family-centered values, may also shape how symptoms are perceived, discussed, and communicated in clinical and online settings [23,24]. Family-centered decision-making and regional differences in access to specialized supportive and palliative services may further influence how symptoms are experienced and communicated in the Chinese context [25-27].

Patient-generated data, defined as health-related data created, captured, or recorded by patients, have considerable potential to improve disease diagnosis and management, enhance health outcomes, and facilitate more connected relationships between patients and health care teams [28]. Previous research has demonstrated that patient-generated data can strengthen patient-provider communication in chronic care settings [29]. During the COVID-19 pandemic, researchers further advocated the use of patient-generated social media data from Twitter (Twitter, Inc) as a real-time source of public health insight, comparable in some respects to large-scale virtual focus groups, to inform responses from health care professionals and governmental authorities [30]. Given the chronic, symptom-intensive, and emotionally complex nature of cancer care, patient-generated narratives may provide particularly valuable insight into how patients experience and communicate illness in everyday contexts. These developments highlight the value of leveraging patient-generated data to better understand how cancer symptoms are experienced and communicated within the Chinese sociocultural context. Such evidence may contribute to more responsive symptom assessment and nursing supportive care strategies.

Evidence from Chinese-language cancer social media studies remains limited, with most analyses focusing on specific cancer types or isolated symptom domains. To address this gap, this study analyzed a large corpus of patient-generated narratives from 2 major Chinese social media platforms to better understand how cancer symptoms are experienced and communicated in naturalistic settings. Specifically, the study examined patterns of SPPADE symptom expression and co-occurrence within patient narratives, explored coping approaches, and identified broader psychosocial and experiential themes through topic modeling. The findings may inform more culturally responsive approaches to oncology symptom assessment and support nursing-led symptom management and supportive care strategies.

## Methods

### Study Design

This study used a computational text analysis integrating large language model (LLM) annotation and neural topic modeling to characterize cancer symptom narratives in Chinese patient-generated social media. The analytical framework combined structured symptom extraction and unsupervised topic modeling to examine both the distribution of predefined symptom dimensions and the psychosocial narrative contexts within which these symptoms were expressed [31]. The research process is illustrated in Figure 1. In addition to the primary analyses of symptom occurrence, co-occurrence, topic modeling, and topic-symptom correspondence, cancer type, disease stage, and coping strategies were examined as supplementary contextual variables to situate the primary findings within relevant clinical and behavioral dimensions.

**Figure 1.**
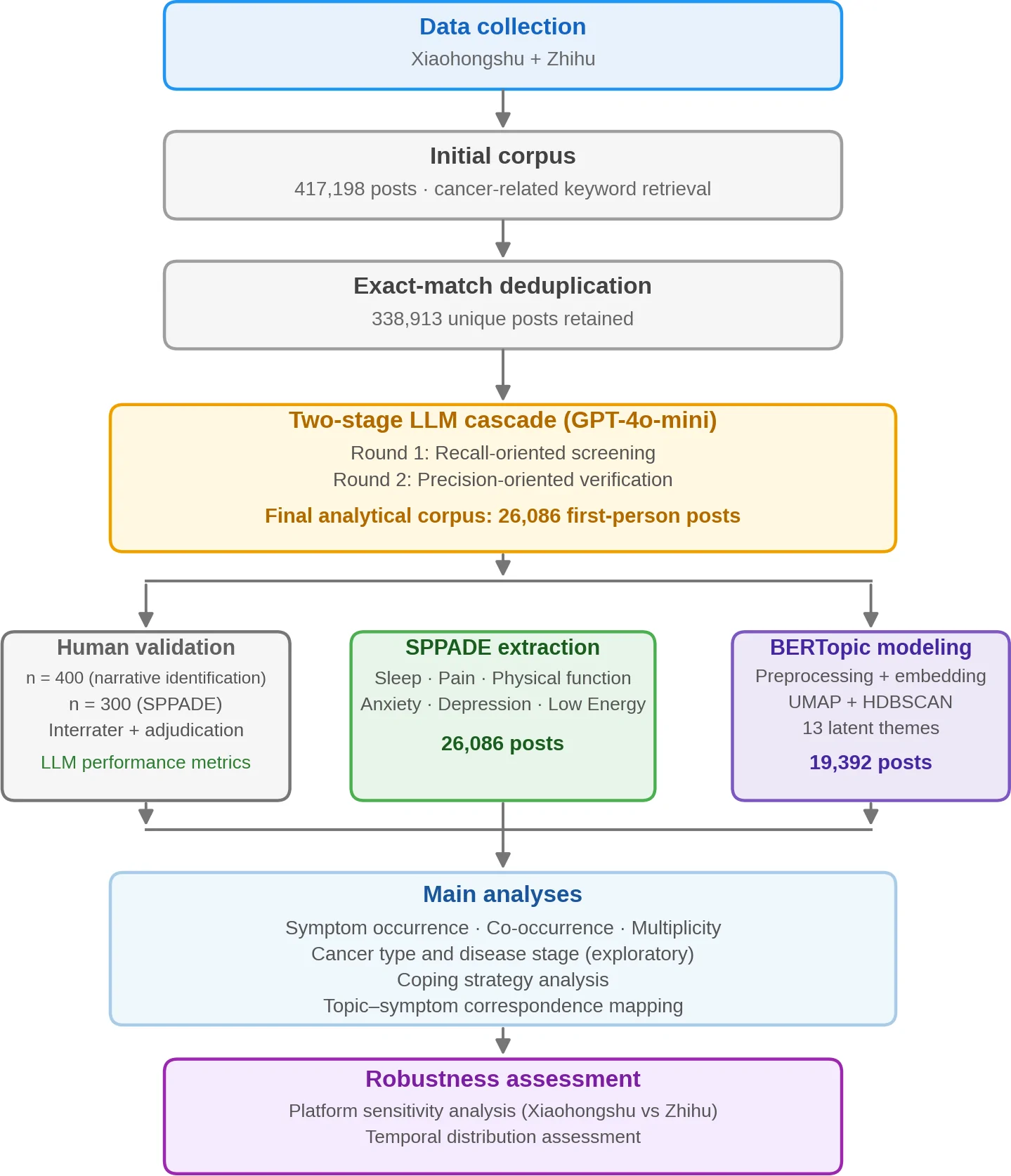
Study design and analytical workflow. HDBSCAN: Hierarchical Density-Based Spatial Clustering of Applications with Noise; LLM: large language model; SPPADE: sleep disturbance, pain, physical function impairment, anxiety, depression, and low energy or fatigue; UMAP: Uniform Manifold Approximation and Projection.

### Data Collection and Preprocessing

#### Platform Selection and Data Acquisition

Data were collected from 2 major Chinese social media platforms: Xiaohongshu and Zhihu. These platforms were selected because they support distinct but complementary forms of patient discourse, potentially capturing a wider range of patient experiences than single-platform datasets [32]. Data collection spanned November 2013 and February 2026, corresponding to the period during which both platforms had reached sufficient user activity to generate substantial cancer-related content. Posts were retrieved using a set of Chinese search terms covering 6 SPPADE symptom domains and patient experience expressions, yielding an initial corpus of 417,198 posts (Xiaohongshu: n=375,658; Zhihu: n=41,540). Full details of the search terms, data collection procedure, and inclusion and exclusion criteria are provided in Multimedia Appendix 1.

#### Deduplication

To eliminate redundant content, exact-match deduplication was applied at the post-content level, whereby posts sharing identical textual content were reduced to a single instance regardless of platform of origin or posting date. This procedure reduced the corpus to 338,913 unique posts.

### LLM-Based Annotation

#### Overview

To structure the large-scale patient-generated social media corpus, a task-specific LLM-based annotation framework was developed. The annotation process consisted of four components: (1) identification of first-person cancer-related patient narratives, (2) extraction of predefined SPPADE symptom domains, (3) identification of cancer type and disease stage, and (4) extraction of coping strategies described in relation to symptom experiences. Task-specific prompts were developed to define the eligibility criteria, annotation rules, and expected output format for each task. Detailed prompts and annotation specifications are provided in Multimedia Appendix 2.

#### First-Person Patient Narrative Identification

To preserve the patient-generated nature of the analytical corpus, we restricted subsequent analyses to posts describing the user’s own cancer-related experience. A 2-round LLM cascade was implemented to identify eligible first-person patient narratives. In the first round, a recall-oriented prompt was applied using GPT-4o-mini (OpenAI) to broadly identify posts containing indications of personal cancer experience, prioritizing sensitivity and minimizing the omission of potentially eligible narratives. In the second round, a precision-oriented prompt was applied to the posts flagged in the first round to determine whether the content represented a genuine first-person patient narrative.

Posts were considered eligible when the user described their own cancer diagnosis, symptoms, treatment, recovery, or related health experiences. Posts primarily describing another person’s experience (eg, a family member or caregiver), general cancer-related information, health education, news, advertising, or other nonexperiential content were excluded. This 2-stage screening process yielded a final corpus of 26,086 first-person cancer-related posts for subsequent analysis. The unit of analysis was the individual social media post rather than the individual patient. Because user identifiers were not available, multiple posts from the same individual could not be linked or deduplicated at the user level. Accordingly, all descriptive estimates represent the proportion of posts containing a given feature and should not be interpreted as patient-level prevalence estimates.

#### SPPADE Symptom Extraction

For each eligible first-person cancer-related post, GPT-4o-mini was used to identify the presence of 6 predefined symptom domains corresponding to the SPPADE framework. The model was instructed to determine whether each symptom was explicitly or contextually described in the patient’s narrative according to predefined operational criteria. Symptom identification was based on the post user’s first-person description of their own symptom experiences, excluding posts in which the user described symptoms experienced by others, provided general medical information, or offered recommendations. Each symptom domain was coded as present or absent using a structured output format to facilitate subsequent quantitative analysis. The 6 symptom domains were selected a priori based on the SPPADE framework and were applied consistently across the full corpus.

#### Cancer Type and Disease Stage Identification

To provide contextual information about the clinical characteristics represented in the social media corpus, cancer type and disease stage were additionally annotated using GPT-4o-mini with task-specific structured prompts (Multimedia Appendices 2 and 3). Exploratory stratified analyses were planned where sufficient information was available to support meaningful comparison. Cancer type was classified according to predefined cancer categories based on information explicitly stated or clearly described in each post. Disease stage was extracted when the patient’s narrative contained sufficient information to permit reliable identification. Posts for which cancer type or disease stage could not be reliably identified were retained in the overall corpus but excluded from the corresponding stratified analyses.

#### Coping Strategy Identification

Because symptom experience also encompasses how patients respond to and manage symptoms, coping strategies described in relation to symptom experiences were additionally characterized. Coping strategies described in relation to cancer-related symptoms were identified using GPT-4o-mini with a structured, symptom-oriented prompt (Multimedia Appendix 2). The model was instructed to identify strategies explicitly described by patients as ways of managing, relieving, or responding to their symptoms or symptom-related experiences. The extracted strategies were subsequently standardized into predefined coping domains informed by established frameworks of cancer symptom management and coping theory [33,34], as specified in the annotation prompt (Multimedia Appendix 2). Multiple coping strategies could be identified within a single post when patients described more than one approach. Because coping-related information was not systematically reported across the corpus, coping-related content was analyzed descriptively to characterize the types of strategies articulated by patients in relation to specific symptom experiences, rather than to estimate the prevalence of coping behaviors at the population level.

### Human Validation of LLM-Based Annotations

#### Validation of First-Person Patient Narrative Identification

To evaluate the reliability of the LLM-based identification of first-person cancer-related patient narratives, a stratified random validation sample of 400 posts was constructed. To ensure adequate representation of both eligible and ineligible posts for evaluating classification performance across both classes, the sample comprised 200 posts classified as eligible and 200 posts classified as ineligible by the LLM. Two trained research assistants with backgrounds in oncology nursing independently reviewed all 400 posts according to the predefined eligibility criteria and classified each post as either a first-person cancer-related patient narrative or a non–first-person narrative. The human raters were blinded to the LLM-generated classifications and to each other’s judgments. Interrater agreement between the two human raters was assessed using percentage agreement and Cohen κ. For posts on which the 2 raters disagreed, a third researcher independently reviewed the discrepant cases and adjudicated the final classification according to the predefined criteria. The resulting adjudicated annotations were treated as the final human reference standard. The performance of the LLM-based classification was then evaluated against the final human reference standard using accuracy, sensitivity, specificity, positive predictive value (PPV), negative predictive value (NPV), *F*_1_-score, and Cohen κ.

#### Validation of SPPADE Symptom Extraction

To assess the reliability of the LLM-based SPPADE symptom extraction, a validation sample was drawn exclusively from posts already classified as eligible, as SPPADE symptom extraction was applied only to confirmed first-person patient narratives. A random sample of 300 first-person cancer-related posts was independently reviewed by 2 trained research assistants. For each post, the raters independently coded the presence or absence of the 6 SPPADE symptom domains using the same predefined operational criteria applied to the LLM annotations. The human annotations were compared independently with the corresponding LLM-generated labels for each symptom domain. Agreement between the LLM and each human rater was assessed using Cohen κ and percentage agreement for each SPPADE symptom domain. The results were used to evaluate the consistency of the LLM-based symptom extraction across the 6 domains.

#### Scope of Human Validation

Human validation was focused on the 2 LLM-based tasks most directly relevant to the primary analytical objectives: first-person patient narrative identification and SPPADE symptom extraction. Cancer type, disease stage, and coping strategy annotations were used as secondary contextual or exploratory variables and were not independently validated against human coding.

### BERTopic-Based Neural Topic Modeling

#### Overview

To identify broader experiential themes beyond predefined SPPADE symptoms, BERTopic, a transformer-based neural topic modeling approach, was applied to cancer-related first-person social media narratives [35,36]. Unlike conventional probabilistic topic models, BERTopic leverages contextual embeddings and density-based clustering to identify semantically coherent topics from short and heterogeneous online texts. After preprocessing, 19,392 posts were retained for topic modeling, whereas 6694 posts were excluded because of limited textual content, excessive noise, or insufficient semantic information for meaningful topic representation [37].

#### Text Preprocessing and Embedding Generation

Chinese text preprocessing included word segmentation using the open-source Python library Jieba, removal of stop words and noninformative symbols, and normalization of domain-specific synonymous expressions. Semantically equivalent expressions related to cancer treatment, symptoms, and health care experiences were standardized to improve topic consistency. Sentence-level semantic representations were generated using the pretrained multilingual sentence-transformer model paraphrase-multilingual-MiniLM-L12-v2, which produces 384-dimensional embeddings.

#### Dimensionality Reduction and Clustering

The generated embeddings were reduced using Uniform Manifold Approximation and Projection (UMAP) with cosine distance. Parameters including n_neighbors and n_components were systematically varied during model optimization to balance local semantic structure and global topic separation. Topic clustering was performed using Hierarchical Density-Based Spatial Clustering of Applications with Noise (HDBSCAN), configured with a Euclidean distance metric, the excess-of-mass (EOM) cluster selection method, min_cluster_size as the main tuning parameter, and prediction data generation enabled. Different values of min_cluster_size were evaluated during model optimization.

#### Topic Model Optimization and Topic Selection

To determine an appropriate topic structure, 18 BERTopic configurations were evaluated through systematic variation of UMAP and HDBSCAN parameters, including n_neighbors, n_components, and min_cluster_size. Candidate models were compared based on topic coherence and topic interpretability, with detailed model configurations and coherence results provided in Multimedia Appendix 4 [38]. Although models with fewer topics occasionally achieved comparable or slightly higher coherence scores, these solutions generated overly broad topics with limited interpretability. The final model was therefore selected by balancing quantitative coherence with qualitative assessment of topic distinctiveness and interpretability.

The selected configuration initially generated 14 topic labels, comprising 13 substantive topics and 1 outlier group (topic –1). Posts initially classified as outliers were subsequently redistributed into the 13 substantive topics using BERTopic’s outlier reduction function. Three reassignment strategies (embedding-based similarity, similarity based on class-based term frequency–inverse document frequency [c-TF-IDF], and topic distribution similarity) were compared, and the embedding-based strategy was adopted as it yielded the highest topic coherence and interpretability. Following outlier reassignment, all posts were assigned to 1 of the 13 final topics, with no remaining unassigned posts.

#### Reproducibility and Final Model Configuration

All analyses were implemented in Python 3.11.13 using BERTopic (version 0.17.4; Maarten Grootendorst), with sentence-transformers (paraphrase-multilingual-MiniLM-L12-v2) for document embeddings. A fixed random seed of 42 was used for the primary analysis, with random_state=42 specified for UMAP. The final model used UMAP with n_neighbors=10 and n_components=2, and HDBSCAN with min_cluster_size=100. Outlier posts were reassigned using the embedding-based similarity strategy. To assess the robustness of the topic solution, a subsequent stability analysis was conducted.

#### Sensitivity and Stability Assessment

To evaluate the robustness of the identified topic structure, a post hoc sensitivity and stability analysis was conducted. First, 112 BERTopic configurations were evaluated through systematic variation of UMAP parameters (n_components [2, 6, 10, 14]; n_neighbors [5, 10, 15, 20]) and HDBSCAN min_cluster_size (25, 50, 75, 90, 100, 125, 150), with candidate configurations ranked by topic coherence (c_v) and cross-seed stability based on pairwise adjusted Rand index (ARI). Second, 12 representative configurations were screened across 3 random seeds (36 runs), with cross-seed agreement assessed using pairwise ARI, normalized mutual information (NMI), and c-TF-IDF keyword overlap. Third, the 2 most stable configurations were evaluated across 11 random seeds (22 runs) for multiseed confirmation. The original 2-dimensional configuration showed substantial sensitivity to random initialization, with pairwise ARI values ranging from 0.06 to 0.11 across seeds, indicating that the exact topic partition was not fully reproducible under varied stochastic conditions. In contrast, the 5-dimensional configuration (n_components=5, n_neighbors=10, and min_cluster_size=75) demonstrated markedly higher cross-seed stability, with a median pairwise ARI of 0.947 across nondegenerate seeds. Importantly, the stable 5-dimensional solution showed substantial correspondence with the original 13-topic structure (ARI=0.808; weighted purity=0.910), suggesting that the major semantic organization captured by the original solution was substantially preserved in the more stable configuration. The complete model selection and stability results are provided in Multimedia Appendix 4, with detailed cluster-to-topic correspondence evidence in Multimedia Appendix 5.

### Topic-Symptom Correspondence Analysis

To examine how predefined SPPADE symptoms were distributed across the broader experiential contexts identified by BERTopic, topic-specific symptom distributions were analyzed following topic assignment. Each post retained both its structured symptom labels and assigned topic label, allowing SPPADE symptom expression to be compared across topics. For each topic, the proportion of posts containing each symptom was calculated. Topic-symptom correspondence was subsequently visualized using a Sankey diagram to illustrate how symptom expression varied across the BERTopic-derived themes.

### Ethical Considerations

This study adhered to the ethical principles of the Declaration of Helsinki and the guidelines of the Association of Internet Researchers [39,40]. Ethical approval was obtained from the Ethics Committee of Anhui Medical University (approval number 81250895). The study involved a noninterventional analysis of publicly accessible content voluntarily posted on Xiaohongshu and Zhihu. No private, restricted, or password-protected content was accessed, and data collection was conducted in accordance with the platforms’ applicable terms of service and policies governing publicly accessible content. Given the use of publicly available, nonidentifiable data and the absence of participant recruitment or interaction, the requirement for individual informed consent was waived by the ethics committee.

To protect user privacy, usernames, user IDs, profile information, and other direct identifiers were not retained in the analytical dataset. Data were deidentified before computational processing, and no attempt was made to identify or contact individual users. Any illustrative quotations included in this paper were deidentified and paraphrased where necessary to minimize the risk of indirect reidentification.

For LLM-based annotation, only deidentified textual content required for computational analysis was processed using GPT-4o-mini. No usernames, user IDs, profile information, or other direct personal identifiers were transmitted to the external LLM service. The processed data were used solely for research purposes and were accessible only to the research team.

## Results

### Validation of LLM-Based Annotations

#### First-Person Patient Narrative Identification

Among the 400 posts included in the validation sample (Tables 1 and 2), the 2 human raters agreed on 357 (89.2%) posts, with a Cohen κ of 0.784 and a Gwet AC1 of 0.787. The raters disagreed on 43 posts, which were subsequently reviewed by a third researcher to establish the final human reference standard. Compared with the adjudicated human reference standard, the LLM achieved an accuracy of 82.2%, sensitivity of 86.4%, specificity of 78.9%, PPV of 76.5%, NPV of 88%, and an *F*_1_-score of 81.2%. The Cohen κ between the LLM and the final human reference standard was 0.645.

**Table 1.** Validation of LLM^a^-based annotations (first-person patient narrative identification)^b^.

Metric		Value
LLM performance against final human reference standard (n=400)		
Accuracy (%)		82.2
Sensitivity (%)		86.4
Specificity (%)		78.9
Positive predictive value (%)		76.5
Negative predictive value (%)		88
*F*_1_-score (%)		81.2
Cohen κ		0.645
Human-human agreement (n=400)		
Agreement, n (%)		357 (89.2)
Cohen κ		0.784
Gwet AC1		0.787
Disagreements, n		43

a LLM: large language model.

b For first-person patient narrative identification, 400 posts were independently assessed by 2 trained research assistants. Disagreements between the human raters (n=43) were adjudicated by a third researcher to establish the final human reference standard.

**Table 2.** Validation of LLM^a^-based annotations (SPPADE^b^ symptom extraction).

SPPADE symptom domain	Rater A versus LLM Cohen κ	Rater B versus LLM Cohen κ
Sleep disturbance	0.897	0.899
Pain	0.764	0.777
Physical function impairment	0.555	0.619
Anxiety	0.66	0.766
Depression	0.697	0.686
Low energy or fatigue	0.712	0.778
Mean κ (SD) across 6 domains	0.714 (0.114)	0.754 (0.095)

a LLM: large language model.

b SPPADE: sleep disturbance, pain, physical function impairment, anxiety, depression, and low energy.

#### SPPADE Symptom Extraction

For SPPADE symptom extraction (Table 2), agreement between the LLM and the 2 human raters was assessed across all 6 symptom domains using Cohen κ. Agreement was highest for sleep disturbance (Cohen κ=0.897 for rater A and 0.899 for rater B) and pain (Cohen κ=0.764 and 0.777, respectively), while physical function impairment showed the lowest agreement (Cohen κ=0.555 and 0.619, respectively). Agreement for anxiety, depression, and low energy or fatigue ranged from Cohen κ=0.660 to 0.778 across the 2 raters. Overall, mean Cohen κ was 0.714 (SD 0.114) for rater A versus the LLM and 0.754 (SD 0.095) for rater B versus the LLM, indicating generally substantial agreement across the 6 symptom domains.

### Symptom Occurrence, Co-Occurrence, and Multiplicity

#### Symptom Occurrence

The final analytical corpus comprised 26,086 posts. The median post length was 36 (IQR 23‐60) characters, reflecting the short-form nature of social media expression. Age and sex were not systematically extractable from post content, with explicit age and sex references identified in only 3.8% (980/26,086) and 1.6% (420/26,086) of posts, respectively. Individual user identifiers were not retained in the analytical dataset; all findings are therefore reported at the post level. Of the 26,086 posts, 13,896 (53.3%) contained at least 1 identifiable SPPADE symptom mention. Pain was the most frequently identified dimension (5783, 22.2%), followed by physical function impairment (4153, 15.9%), depression (4050, 15.5%), anxiety (2973, 11.4%), low energy or fatigue (2048, 7.9%), and sleep disturbance (1955, 7.5%).

#### Symptom Co-Occurrence

The symptom co-occurrence matrix showed that pain and physical function impairment formed the most frequent co-occurring pair (n=1407), followed by pain and depression (n=1024), physical function impairment and low energy or fatigue (n=993), depression and anxiety (n=837), and pain and sleep disturbance (n=762; Figure 2). Cell values represent the number of posts in which both symptoms were co-identified. The diagonal displays the total count of posts containing each symptom. Color intensity reflects co-occurrence frequency. Across all symptom dimensions, pain showed the strongest overall co-occurrence pattern, followed by physical function impairment.

**Figure 2.**
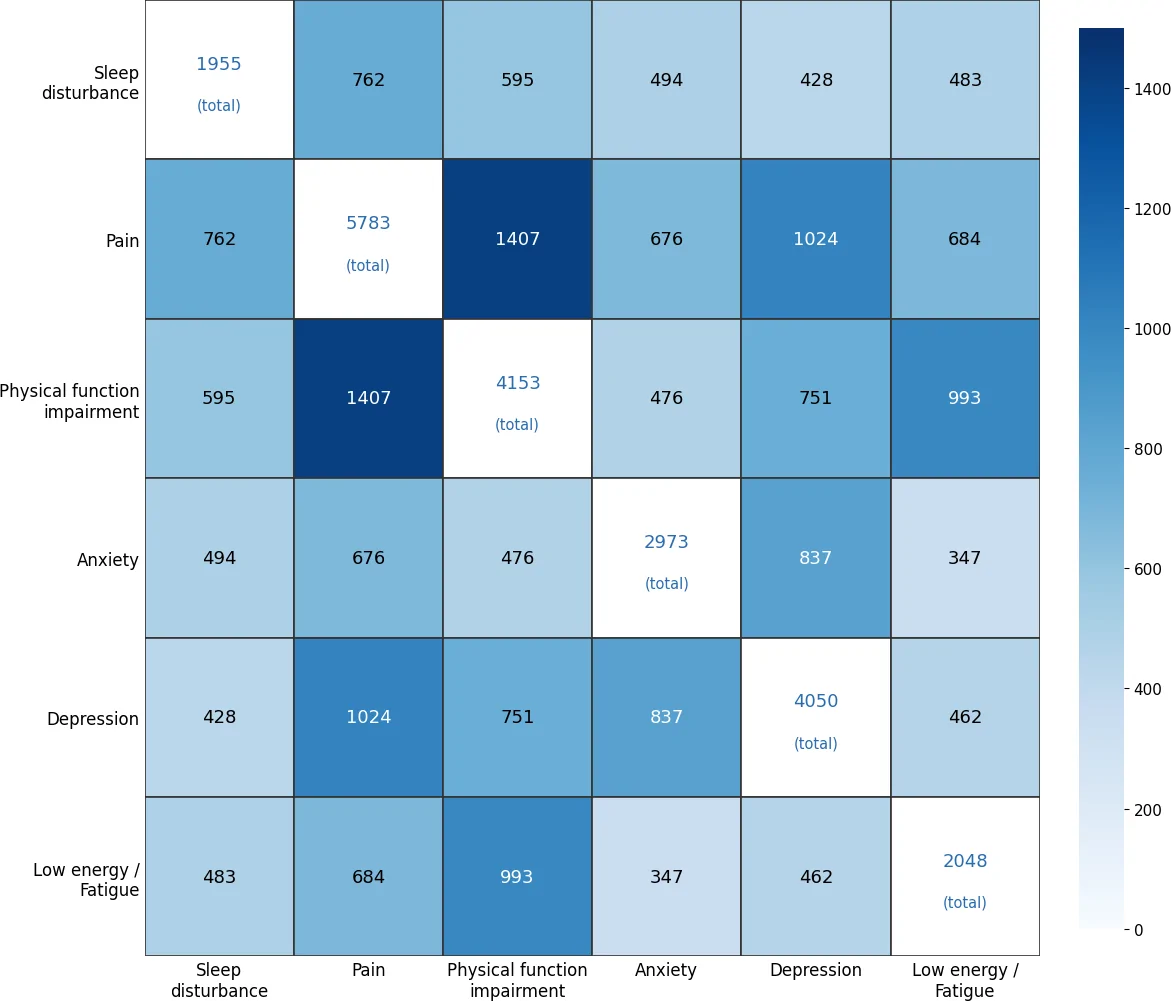
Symptom co-occurrence matrix among posts with at least 1 SPPADE (sleep disturbance, pain, physical function impairment, anxiety, depression, and low energy or fatigue) symptom.

#### Symptom Multiplicity

Among the 26,086 posts, 46.7% (n=12,190) contained no identifiable SPPADE symptoms, 34.5% (n=8990) contained exactly 1 symptom mention, 13.4% (n=3506) contained 2 co-occurring symptom mentions, and 5.4% (n=1400) contained 3 or more co-occurring symptom mentions, indicating that most posts reflected limited symptom multiplicity while a smaller subset contained substantial multisymptom expression.

### Clinical Contextual Variation in Symptom Profiles

#### Cancer Type Distribution

Cancer type was identified in 3197 (12.3%) posts; posts without identifiable cancer type were retained in the full corpus but excluded from stratified analyses. Lung cancer was the most frequently represented diagnosis (805/3197, 25.2%), followed by breast (403/3197, 12.6%), colorectal (363/3197, 11.4%), liver (320/3197, 10%), and gastric cancer (314/3197, 9.8%), together accounting for 69% (2205/3197) of identified cases.

#### Symptom Profiles by Cancer Type

Symptom profiles varied across cancer types (Figure 3). Cell values represent the percentage and count of posts within each cancer type in which the symptom was identified; color intensity corresponds to the percentage, and cancer types are ranked by total post volume; red borders indicate the cells with the highest proportion of posts containing that symptom within each cancer type row. Lung and gastric cancer posts showed the highest proportions of posts containing physical function impairment and pain. Breast cancer posts were characterized by higher proportions of posts containing depression (93/403, 23.1%) and anxiety (86/403, 21.3%), whereas colorectal cancer showed a more evenly distributed profile. Liver cancer posts showed comparatively lower proportions of posts containing anxiety and sleep disturbance.

**Figure 3.**
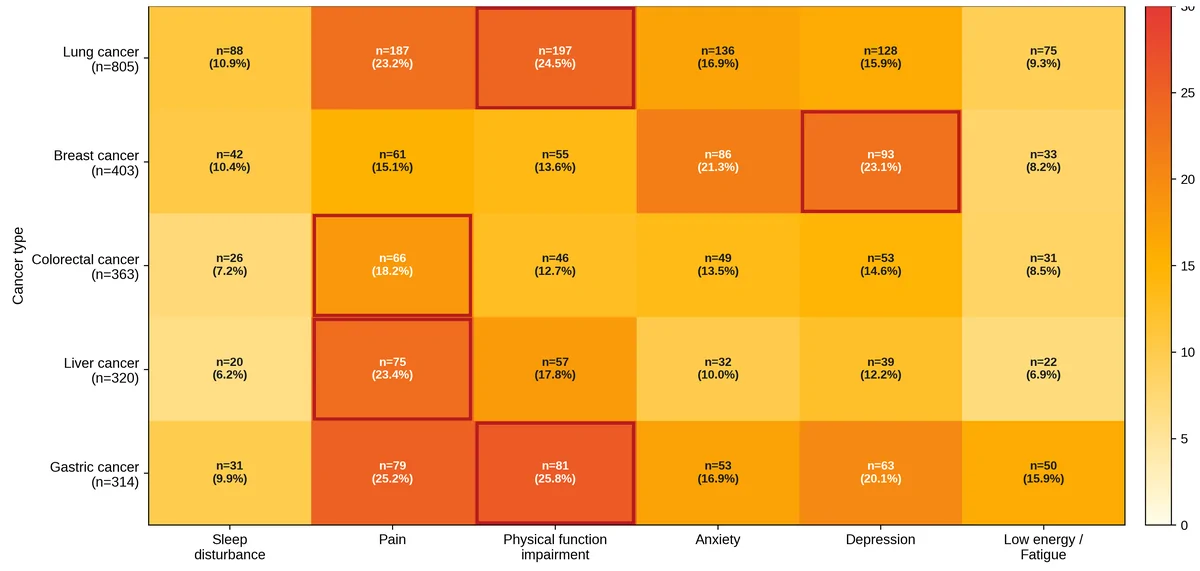
Symptom occurrence across the 5 most common cancer types.

#### Symptom Profiles by Disease Stage

Disease stage was ascertainable in 2629 (10.1%) posts, of which 2518 were classified as advanced stage and 111 as early stage. Given the limited ascertainment and substantial group imbalance, stage-stratified symptom analyses were not pursued further.

#### Coping Strategy

Coping strategies were identified in 7104 posts (27.2% of the corpus), reflecting the subset of patient narratives in which symptom management approaches were explicitly described. Medication-based coping was the most prominently expressed strategy, particularly in posts describing pain and sleep disturbance. Social support and psychological support featured prominently in posts describing anxiety and depression, suggesting that patients experiencing emotional symptoms were more likely to articulate needs for interpersonal and psychological resources. Physical function impairment was frequently accompanied by expressions of medical support-seeking, while posts describing low energy or fatigue more commonly referenced lifestyle-based approaches such as rest, dietary adjustment, and activity modification.

#### Model Selection and Topic Solution

Model selection was guided by a combination of semantic coherence and topic interpretability. Across 18 candidate configurations, coherence scores ranged from 0.5068 to 0.5829 (Multimedia Appendix 4). The selected solution (n_components=2, n_neighbors=10, and min_cluster_size=100) initially generated 14 topic labels, comprising 13 substantive topics and 1 outlier group (topic –1), to which 2948 (15.2%) posts were assigned by HDBSCAN. To maximize corpus coverage, outlier posts were subsequently redistributed into the 13 substantive topics using the reduce_outliers procedure. Three reassignment strategies (embedding-based similarity, c-TF-IDF similarity, and topic distribution similarity) were compared, and the embedding-based strategy yielded the highest postreassignment coherence (c_v=0.5975) and was therefore adopted. Following outlier reassignment, all 19,392 posts were assigned to one of the 13 final topics, with no remaining unassigned posts. A subsequent sensitivity analysis confirmed that while the original 2-dimensional configuration was sensitive to random initialization, the major semantic structure of the 13-topic solution was robust, with a stable alternative configuration showing high correspondence to the original topics (ARI=0.808; weighted purity=0.910; Multimedia Appendices 4 and 5).

#### Topic Structure and Thematic Characteristics

BERTopic analysis identified 13 latent themes within the 19,392 patient-generated posts (Table 3; Multimedia Appendix 6). Topics are labeled T0 to T12 following BERTopic’s internal cluster indexing. Topic sizes ranged from 160 (0.8%; T3: financial burden and access to treatment) to 7200 (37.1%; T4: cancer treatment trajectory and therapeutic strategies) posts, indicating substantial variation in thematic prominence across patient-generated narratives, with treatment trajectory and therapeutic decision-making constituting the dominant narrative context. To facilitate interpretation of topic semantics, c-TF-IDF scores were calculated for representative terms within each topic, with higher scores indicating greater term importance.

**Table 3.** BERTopic themes, representative keywords, and descriptions.

Topic	Top keywords (c-TF-IDF^a^)	Description
T0: Family involvement and care decision-making	family member (家属); health care provider (医护人员); in situ (原位); cancer (癌症); hospital (医院); life (人生)	Captured the central role of family in the cancer experience, encompassing the emotional, practical, and financial dimensions of family involvement in care. The narratives described concerns about concealing diagnoses from family members and feelings of guilt about imposing caregiving demands on loved ones.
T1: Liver metastasis and hepatic dysfunction	suprahepatic (肝上); hepatic metastasis (肝转); liver function (肝功能); liver (肝脏); cirrhosis (肝硬化); metastasis (转移)	Captured the clinical trajectory of hepatic involvement, including monitoring of liver function indices, management of jaundice and ascites, and progressive loss of functional independence associated with hepatic failure.
T2: Diagnostic process and anticipatory anxiety	tomorrow (明天); discharge (出院); examination (检查); enhanced CT^b^ (增强CT); MRI^c^ (核磁); plan (方案)	Captured acute anticipatory distress concentrated at diagnostic decision points, distinct from the sustained psychological distress of T6. The narratives described uncertainty while awaiting test results and difficulties planning beyond the immediate future.
T3: Financial burden and access to treatment	cost (费用); medical insurance (医保); hospital (医院); treatment (治疗); imported (进口); surgery (手术)	Captured the substantial financial burden of cancer treatment in the Chinese health care context, including out-of-pocket expenditure, limitations of medical insurance coverage, and difficult trade-offs between treatment continuation and financial sustainability.
T4: Cancer treatment trajectory and therapeutic strategies	chemotherapy (化疗); cancer (癌症); surgery (手术); advanced stage (进展期); metastasis (转移); immunotherapy (免疫治疗)	The largest topic in the corpus, capturing a broad range of therapeutic modalities including surgery, chemotherapy, targeted therapy, immunotherapy, and radiotherapy. The narratives described treatment trajectories, including treatment responses, side-effect management, and decisions regarding treatment continuation or cessation.
T5: Sleep disturbance with day-night variation	sleep (睡眠); day-night variation (昼夜感受); sleeping (睡觉); discomfort (不适); confusion (意识模糊); fatigue (疲乏)	Captured chronic insomnia, frequent nocturnal awakenings, and disruption of the day-night cycle extending beyond treatment-related side effects. The narratives portrayed sleep disturbance as highly distressing and, in some cases, as more burdensome than other aspects of the illness, with associated psychological distress.
T6: Psychological distress: anxiety and depression	anxiety (焦虑); family member (家属); depression (抑郁); discomfort (不适); life (人生); external support (外界支持)	Capturing co-occurring fear, despair, and emotional suppression. The narratives included accounts of concealing distress from family members alongside expressions of hopelessness, reflecting the coexistence of psychological distress and concerns about its social expression.
T7: Opioid use and pain management	morphine (吗啡); medication (用药); fentanyl (芬太尼); pain relief (止痛); oxycodone (羟考酮); side effects (副作用)	Captured narratives concerning cancer pain and opioid-based analgesia in advanced disease. The narratives described increasing analgesic requirements, concerns about medication effectiveness, and challenges in achieving adequate pain control.
T8: Health care interaction and treatment experience	surgery (手术); healthcare provider (医护人员); hospital (医院); treatment (治疗); metastasis (转移); examination (检查)	Captured narratives concerning navigation of the health care system, including interactions with clinicians, decisions about surgical intervention, and challenges of accessing appropriate care. The narratives reflected the complexity of treatment decision-making and, in some cases, concerns about communication and perceived lack of compassion in clinical encounters.
T9: Death-related cognition and existential distress	life (人生); discomfort (不适); death (死亡); resilience (韧性); will to survive (求生欲); hope (希望)	Captured the existential dimension of the cancer experience, encompassing confrontation with mortality, the search for meaning, and the oscillation between despair and resilience. Narratives described the preparation of advance directives and the process of accepting a terminal prognosis.
T10: Appetite loss, nutrition, and physical decline	appetite problem (食欲问题); confusion (意识模糊); nutrition (营养); discomfort (不适); eating (吃饭); symptom (症状)	Captured narratives concerning reduced appetite, nutritional difficulties, and physical deterioration, particularly in the context of treatment-related gastrointestinal symptoms. The narratives described reduced oral intake, weight loss, and distress associated with physical decline.
T11: Metastasis and physical manifestations	body site (躯体部位); metastasis (转移); body (身体); ascites (腹水); discovery (发现); lymph node (淋巴结)	Captured the somatic consequences of metastatic spread, including the discovery of new metastatic sites and accumulation of disease-related physical complications. Narratives conveyed the shock of rapid disease progression and its impact on functional capacity.
T12: Dyspnea and respiratory support	cough (咳嗽); breathing (呼吸); fever (发烧); lung (肺部); dyspnea (呼吸困难); throat (喉咙)	Captured dyspnea and respiratory compromise as prominent physical concerns within advanced cancer narratives. The narratives described severe breathlessness that interfered with basic activities and, in some cases, was accompanied by existential concerns.

a c-TF-IDF: class-based term frequency–inverse document frequency.

b CT: computed tomography.

c MRI: magnetic resonance imaging.

#### Narrative Characteristics of Identified Topics

The 13 identified topics reflected multiple dimensions of cancer symptom experience and illness-related concerns expressed in patient-generated narratives (Table 3 and Multimedia Appendix 6). Several topics were centered on embodied physical symptom experiences. T7 (opioid use and pain management, 360/19,392, 1.9%) captured narratives describing refractory pain, opioid escalation, and concerns regarding analgesic effectiveness. T10 (appetite loss, nutrition, and physical decline, 1364/19,392, 7%) reflected severe nutritional compromise, weight loss, and progressive functional deterioration. T12 (dyspnea and respiratory support, 484/19,392, 2.5%) focused on respiratory distress and oxygen dependence, while T11 (metastasis and physical manifestations, 497/19,392, 2.6%) and T1 (liver metastasis and hepatic dysfunction, 254/19,392, 1.3%) described symptom experiences associated with metastatic progression and organ dysfunction.

Psychological concerns were primarily represented by 2 topics. T6 (psychological distress: anxiety and depression, 2097/19,392, 10.8%) captured sustained emotional burden characterized by hopelessness, emotional concealment, and co-occurring anxiety and depression. In contrast, T2 (diagnostic process and anticipatory anxiety, 165/19,392, 0.9%) reflected acute anxiety surrounding diagnostic uncertainty, treatment decision-making, and fear of disease progression. Sleep-related experiences were represented by a distinct topic, T5 (sleep disturbance with day-night variation, 853/19,392, 4.4%), which described persistent insomnia and disrupted sleep rhythms, often accompanied by emotional exhaustion and worsening physical discomfort.

In addition to symptom-focused narratives, several of the most prominent topics reflected broader experiential contexts surrounding cancer care and illness management. T4 (cancer treatment trajectory and therapeutic strategies, 7200/19,392, 37.1%) was the most prominent topic and centered on treatment planning, chemotherapy experiences, and therapeutic decision-making. T8 (health care interaction and treatment experience, 4104/19,392, 21.2%) captured interactions with health care professionals and perceptions of clinical care. Other topics reflected existential confrontation with mortality (T9: death-related cognition and existential distress, 1344/19,392, 6.9%), family involvement and caregiving burden (T0: family involvement and care decision-making, 510/19,392, 2.6%), and financial toxicity related to treatment access and affordability (T3: financial burden and access to treatment, 160/19,392, 0.8%).

#### Topic-Symptom Correspondence

Topic-specific symptom distributions varied substantially across the 13 BERTopic-derived themes (Figure 4 and Multimedia Appendix 7). In the diagram, left-side theme nodes are displayed at equal height, and ribbon width represents the proportion of posts within each theme in which the corresponding symptom was identified, with wider ribbons indicating a higher within-theme proportion. Right-side symptom node height represents the total number of posts containing each symptom across all 13 themes. Ribbon widths at the 2 sides are scaled independently, representing within-theme symptom proportions and overall symptom counts, respectively; the diagram shows correspondence and does not represent conserved flow between the two sides. Ribbon colors correspond to the originating BERTopic theme. T5 (sleep disturbance with day-night variation) showed the highest proportion of posts describing sleep disturbance (591/853, 69.3%), T7 (opioid use and pain management) showed the highest proportion of posts describing pain (224/360, 62.2%), and T6 (psychological distress: anxiety and depression) demonstrated higher proportions of posts describing both anxiety (619/2097, 29.5%) and depression (729/2097, 34.8%).

**Figure 4.**
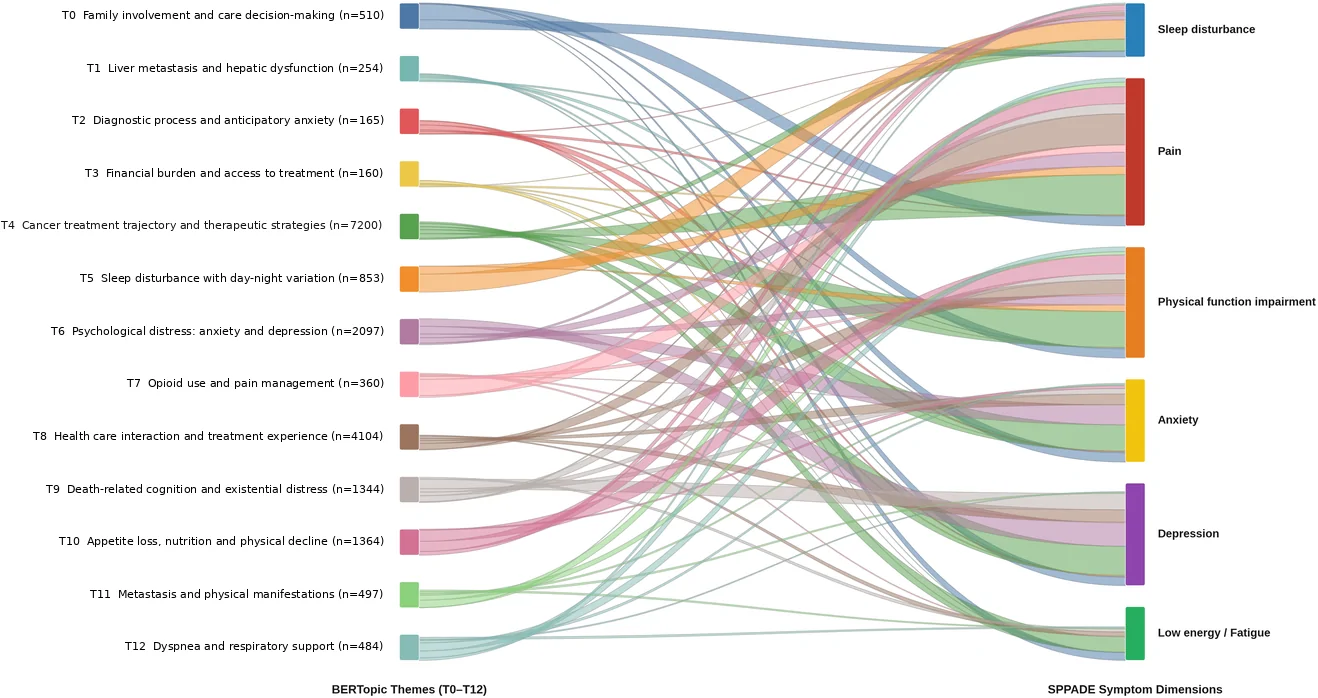
Sankey-style diagram of the correspondence between BERTopic-derived themes and SPPADE (sleep disturbance, pain, physical function impairment, anxiety, depression, and low energy or fatigue) symptom dimensions.

Several topics exhibited higher symptom proportions across multiple symptoms. T0 (family involvement and care decision-making) showed consistently high proportions of posts across all 6 symptoms, ranging from 35.9% to 59.8%. In contrast, T3 (financial burden and access to treatment) and T1 (liver metastasis and hepatic dysfunction) showed comparatively lower proportions across all symptom dimensions.

Overall, symptoms were distributed across multiple thematic contexts rather than concentrated within isolated topics, indicating substantial overlap between symptom expression and broader experiential narratives.

#### Platform Comparison and Temporal Distribution

Given the use of 2 social media platforms with distinct discourse characteristics and unequal contributions to the corpus, symptom distributions were compared across platforms to examine the extent to which the observed patterns varied by platform composition. In addition, because the dataset spanned a long collection period, the temporal distribution of posts was examined to characterize the periods most strongly represented in the corpus.

Symptom proportions were compared between Xiaohongshu (23,396/26,086, 89.7%) and Zhihu (2690/26,086, 10.3%). The overall proportion of posts containing at least 1 SPPADE symptom was comparable between platforms (53.2% vs 54.1%; chi-square test, *P*=.35), suggesting that the overall symptom occurrence was relatively robust to platform composition. Physical function impairment also showed no significant difference between platforms (15.9% vs 16.0%; *P*=.89). However, Zhihu posts had significantly higher proportions of anxiety (21.7% vs 10.2%; *P*<.001), depression (25.2% vs 14.4%; *P*<.001), low energy or fatigue (10.9% vs 7.5%; *P*<.001), and sleep disturbance (9.3% vs 7.3%; *P*<.001), while the difference in pain was smaller but statistically significant (23.9% vs 22.0%; *P*=.02). These findings indicate that although the overall proportion of posts containing SPPADE symptoms was similar across platforms, the distribution of individual symptom domains varied by platform.

The temporal distribution of the analytical corpus was highly uneven across the study period. Posting volume increased from 655 posts in 2023 to 2696 in 2024 and 16,943 in 2025, with 4363 posts collected through February 2026, and the remaining 1429 (5.5%) posts from 2022 and earlier. The corpus was predominantly composed of posts from 2025 (16,943/26,086, 65%), whereas substantially fewer posts were available from earlier years.

## Discussion

### Principal Findings

This study integrated large-scale symptom extraction and neural topic modeling to examine how cancer symptoms are expressed within patient-generated narratives on Chinese social media platforms. Three principal findings emerged. First, pain and physical function impairment constituted the most prominently expressed and co-occurring symptom dimensions, while sleep disturbance and fatigue were relatively underrepresented despite their known clinical burden, suggesting that social media narratives may selectively foreground symptoms perceived as acute or requiring immediate action. Second, symptom expression was embedded within broader narrative contexts rather than expressed as isolated clinical complaints: topic modeling identified 13 thematic structures spanning treatment decision-making, health care interaction, family involvement, existential distress, and financial burden, within which symptoms were differentially distributed. Third, the correspondence between symptom dimensions and narrative themes revealed that distinct illness contexts were associated with qualitatively different symptom profiles, indicating that patient-generated narratives may capture experiential dimensions not fully represented in conventional structured symptom assessment. Together, these findings demonstrate the value of integrating structured symptom characterization with narrative-based computational approaches to better understand the multidimensional nature of cancer symptom experience [41].

### Selective Symptom Expression in Patient-Generated Narratives

The symptom occurrence hierarchy observed in this study diverged substantially from patterns reported in large-scale clinical patient-reported outcome (PRO) studies. In the E2C2 program [14], a cluster-randomized pragmatic trial enrolling 50,207 oncology patients across 15 clusters of medical oncology and hematology clinics, baseline electronic patient-reported outcome measure (ePROM) scores on a 0‐10 Numeric Rating Scale (NRS) in the usual care group indicated that fatigue was the most severely rated SPPADE dimension (mean 3.17, SD 2.54), followed by sleep disturbance (mean 2.57, SD 2.52), impaired physical function (mean 2.44, SD 2.45), anxiety (mean 2.24, SD 2.35), pain (mean 2.15, SD 2.38), and depression (mean 1.91, SD 2.23). In contrast, pain was the most frequently mentioned symptom in the present patient-generated narrative corpus (5783/26,086, 22.2%), while fatigue (2048/26,086, 7.9%) and sleep disturbance (1955/26,086, 7.5%) were relatively underrepresented. These 2 bodies of evidence are not directly comparable: clinical PRO studies measure symptom severity using standardized numerical rating scales administered at clinical encounters, whereas this study captures the frequency with which symptoms are spontaneously expressed in naturalistic online narratives. This divergence therefore suggests that symptom occurrence patterns within patient-generated text may reflect selective expression dynamics rather than direct correspondence with clinical symptom burden.

One possible interpretation is that symptoms perceived as acute, disruptive, or requiring immediate attention may be more likely to be articulated in online narratives. Pain narratives frequently centered on inadequate analgesia, opioid escalation, and failed symptom control attempts. This was particularly evident within T7 (opioid use and pain management), characterized by keywords including morphine, fentanyl, oxycodone, and pain relief (Table 3), and by representative patient narratives such as “The whole body hurts. The oral medication is sustained-release morphine, 5 tablets now and still no effect” and “Only a few days left to live, who cares about addiction? Even if it causes addiction, if it means going peacefully, it’s worth it” (Multimedia Appendix 6). In this context, pain was not merely described as a bodily sensation, but as an unresolved clinical and existential problem requiring negotiation, intervention, and urgent attention. This action-oriented pattern may partly account for the prominence of pain despite its comparatively lower severity ratings in conventional oncology PRO datasets such as E2C2 [12,14,42].

Nevertheless, BERTopic analysis demonstrated that when sleep disturbance was discussed, it was often described as profoundly distressing. Sleep disturbance was mentioned in 7.5% (1955) of posts, and T5 (sleep disturbance with day-night variation), characterized by keywords including sleep, day-night variation, discomfort, and fatigue (Table 3), captured narratives of persistent insomnia, circadian disruption, emotional exhaustion, and prolonged nocturnal suffering, as illustrated by representative accounts such as “No matter day or night, I just can’t sleep. It’s so painful. Even sleeping pills can’t match a normal person’s sleep. The cancer cells haven’t killed me, but the sleep deprivation nearly has” (Multimedia Appendix 6). These findings suggest that low narrative frequency should not be interpreted as low experiential burden. The co-occurrence of sleep disturbance with pain narratives in this corpus is consistent with evidence that sleep disruption and pain form a mutually reinforcing cycle, whereby pain impairs sleep architecture and sleep deprivation in turn heightens pain sensitivity [43]. By contrast, chronic or gradually accumulating symptoms appeared less likely to be explicitly articulated [44]. Fatigue and sleep disturbance, although common in clinical oncology populations, were relatively infrequently reported within the present corpus. This underexpression may reflect the normalization of persistent suffering during cancer treatment, whereby symptoms perceived as inevitable consequences of illness or therapy become incorporated into everyday life and therefore less likely to prompt active disclosure [45-47].

### Contextualized Symptom Experience in Patient-Generated Narratives

The present findings suggest that cancer symptoms in patient-generated narratives were rarely expressed as isolated bodily sensations. Rather, symptom experiences were embedded within illness narratives involving treatment decision-making, health care interactions, family responsibility, and confrontation with mortality. This narrative contextualization may help explain why substantial overlap was observed between symptoms and multiple BERTopic-derived themes, rather than a discrete one-to-one correspondence between individual symptoms and isolated thematic categories.

Treatment-related narratives formed the dominant thematic context within the corpus, encompassing both therapeutic decision-making and health care system navigation. Symptoms were not expressed as independent experiences within these narratives, but as consequences, indicators, or trade-offs embedded within broader processes of treatment selection, adverse effect management, and clinical communication [48-50]. This was evident in the topic-symptom correspondence patterns observed across T4 (cancer treatment trajectory and therapeutic strategies; 37.1%, 7200/19,392, of posts) and T8 (health care interaction and treatment experience; 21.2%, 4104/19,392, of posts), where pain, physical function impairment, anxiety, and depression were all substantially represented despite neither of these topics being defined by symptom content per se (Multimedia Appendix 7). Narratives such as “Our local hospital told us to take targeted drugs, but they’re not covered by insurance. We went to 2 hospitals in Shanghai and they said no intervention was needed” (Multimedia Appendix 6) illustrate how symptom distress and systemic care barriers were experienced as inseparable. These findings suggest that patients interpreted symptom burden through the broader lens of therapeutic decision-making and health care access, rather than as discrete bodily events [51-54].

Beyond treatment contexts, the same symptom dimensions were expressed across qualitatively distinct narrative themes. Physical function impairment, for example, appeared across T10 (appetite loss, nutrition, and physical decline; 7%, 1364/19,392, of posts), T12 (dyspnea and respiratory support; 2.5%, 484/19,392), T1 (liver metastasis and hepatic dysfunction; 1.3%, 254/19,392), and T11 (metastasis and physical manifestations; 2.6%, 497/19,392), reflecting progressive bodily decline, organ dysfunction, and respiratory compromise as distinct but overlapping experiential trajectories. Psychological symptoms similarly spanned multiple contexts, with anxiety and depression prominent in T6 (psychological distress: anxiety and depression; 10.8%, 2097/19,392) and anticipatory distress emerging distinctly in T2 (diagnostic process and anticipatory anxiety; 0.9%, 165/19,392), as captured by “I don’t dare to make plans for anything more than 2 weeks away. Anything further than that, I don’t even dare think about it” (Multimedia Appendix 6). These patterns indicate that structured symptom extraction identifies what symptoms are present, while topic modeling reveals the diverse experiential contexts within which those symptoms are embedded [55-58].

Family involvement also emerged as a major contextual layer. T0 (family involvement and care decision-making; 2.6%, 510/19,392, of posts) showed relatively high proportions of posts containing each of the 6 SPPADE symptoms, indicating broad overlap between family-centered narratives and symptom expression. Symptom experiences were frequently described alongside concerns about caregiving burden, treatment affordability, and emotional concealment within family relationships, suggesting that symptoms were often narrated relationally rather than individually in this population [59-63].

Existential confrontation with mortality represented a further distinct narrative context. T9 (death-related cognition and existential distress; 6.9%, 1344/19,392 of posts) captured narratives involving fear of death, meaninglessness, and the oscillation between despair and resilience, as illustrated by “Honestly, right now I don’t know whether it’s better to be alive or to be gone. It’s so tormenting” (Multimedia Appendix 6). These narratives encompassed forms of suffering, including mortality awareness and the search for meaning, that extend beyond conventional anxiety and depression constructs, suggesting that patient-generated narratives may capture experiential dimensions that complement standardized symptom assessment [64,65].

### Coping Patterns in Patient-Generated Narratives

Medication-based coping was the most prominently articulated approach in the social media narratives, particularly in posts describing pain and sleep disturbance. Coping expressions also varied across symptom domains: social and psychological support featured more commonly in narratives involving anxiety and depression, whereas lifestyle-based approaches were more evident in relation to fatigue.

The prominence of medication-based coping is noteworthy given the emphasis on multimodal symptom management in supportive cancer care, where behavioral, rehabilitative, and psychosocial approaches are often recommended alongside pharmacological treatment [66-68]. However, the relative prominence of medication-based coping in online narratives should not be interpreted as evidence of limited use or availability of nonpharmacological approaches in clinical or everyday settings. Social media discourse may preferentially foreground active help-seeking and immediate symptom-relief efforts, making medication-related strategies more visible. Future research using structured coping assessments, prospective surveys, or qualitative interviews could examine how closely online coping narratives reflect patients’ broader coping practices and identify opportunities to strengthen awareness of and access to multimodal symptom management support.

### Implications for Narrative-Informed Symptom Assessment

The present findings suggest that patient-generated narratives may provide important complementary perspectives to conventional symptom assessment approaches in oncology care. While structured symptom frameworks such as SPPADE enable systematic identification and quantification of clinically relevant symptoms, patient narratives contextualize how these symptoms are experienced, interpreted, and negotiated within everyday illness trajectories. SPPADE symptom expressions occurred across thematic contexts involving treatment decision-making, health care interaction, family involvement, and existential concerns. These findings suggest that understanding how symptoms are expressed may benefit from attention to the broader experiential contexts in which patients discuss them.

The study also highlights the importance of recognizing selective symptom expression in patient-generated discourse. Symptoms requiring urgent management or immediate action, such as pain and respiratory distress, appeared highly visible within narratives, whereas chronic or normalized symptoms, including fatigue and sleep disturbance, appeared comparatively underarticulated despite their known clinical burden. This discrepancy suggests that narrative visibility should not be interpreted as a direct proxy for symptom severity. Instead, patient-generated narratives may reflect the interaction between symptom burden, help-seeking motivation, sociocultural norms, and perceived treatability.

From a nursing perspective, these findings support the value of integrating narrative-informed approaches into symptom assessment and supportive care. Structured symptom measures are designed to assess predefined symptom domains and may therefore not encompass broader concerns such as family burden, health care navigation, and existential distress, all of which emerged prominently within patient narratives. Incorporating narrative-based information could potentially support more person-centered assessment and help clinicians recognize supportive care concerns that may not be readily captured through structured symptom measures alone.

Future research should examine whether and how narrative information adds clinically useful information to established PROMs. Prospective and longitudinal patient-level studies could explore changes in symptom narratives across the illness trajectory, while implementation-oriented research is needed to assess the feasibility, acceptability, and clinical utility of incorporating narrative information into routine symptom assessment and supportive care.

### Strengths and Limitations

This study has several notable strengths. First, the study analyzed a large corpus of 26,086 first-person cancer-related social media posts, representing one of the largest analyses of Chinese cancer-related social media narratives to date, enabling large-scale examination of naturally expressed cancer-related symptom experiences and concerns that would be difficult to capture through conventional survey-based approaches alone. Second, the integration of structured LLM-based symptom extraction with unsupervised neural topic modeling enabled both systematic characterization of predefined symptoms and inductive identification of emergent experiential themes. This combined analytical strategy provided complementary perspectives on patient experience beyond what either approach could achieve independently. Third, the inclusion of 2 major Chinese social media platforms provided complementary sources of patient-generated discourse and allowed the robustness of symptom findings to be examined across platforms. Fourth, the LLM annotation protocol demonstrated generally substantial agreement with human raters across the 6 SPPADE symptom dimensions, with mean Cohen κ values of 0.714 and 0.754 for the 2 raters, respectively, supporting the feasibility of automated large-scale symptom annotation in Chinese patient-generated narratives.

Several limitations should also be acknowledged. First, the representativeness of the social media corpus is limited. Social media users are unlikely to be representative of the broader cancer population, as platform participation depends on digital access, platform use, and willingness to publicly share illness experiences. In particular, older patients, those living in rural areas, and those with lower income or limited digital literacy are likely underrepresented in the corpus, and their symptom experiences and concerns may differ systematically from those captured here. In addition, the corpus was substantially larger on Xiaohongshu than on Zhihu. Although sensitivity analysis showed that the overall proportion of posts containing at least 1 SPPADE symptom was similar across platforms, differences in individual symptom distributions indicate that platform composition may still influence specific symptom occurrence estimates. These factors limit the generalizability of symptom occurrence estimates and thematic distributions to the wider cancer population. Second, the available clinical contextual information and study design constrain interpretation. Cancer type and disease stage could be identified in only a minority of posts, limiting the interpretability of stratified analyses, which should therefore be regarded as descriptive rather than epidemiologically representative. The cross-sectional nature of the dataset also precludes causal or longitudinal inference. Although the corpus spans 2013‐2026, its temporal distribution was highly uneven, with most posts originating from 2025. The findings therefore primarily reflect more recent online discourse rather than an evenly distributed longitudinal sample, and temporal changes in cancer care practices, treatment availability, and online discourse may have influenced the observed narratives. Furthermore, the absence of linked clinical assessment data precludes direct comparison between narrative-derived symptom patterns and structured patient-reported outcomes, limiting the extent to which the complementary value of social media narratives can be empirically demonstrated. Future research linking patient-generated narratives with clinical PRO data would help establish the incremental validity of narrative-based approaches in oncology symptom assessment. Third, LLM-based annotation introduces measurement uncertainty. Although the validation results demonstrated generally substantial agreement with human raters, agreement was lower for physical function impairment than for several other SPPADE domains, suggesting greater uncertainty when symptoms are expressed indirectly through functional descriptions. Furthermore, cancer type, disease stage, and coping strategy annotations were used as secondary or exploratory variables and were not independently validated against human coding, and their accuracy should therefore be interpreted with caution. Fourth, the topic modeling component is subject to stochastic variation inherent in the UMAP-HDBSCAN pipeline. Sensitivity analysis indicated that the original 2-dimensional UMAP configuration was sensitive to random initialization, with cross-seed agreement substantially lower than that observed for higher-dimensional configurations. Although a stable alternative configuration yielded a topic solution with high correspondence to the original 13-topic structure (ARI=0.808; weighted purity=0.910), the exact topic boundaries should be interpreted as a data-driven exploratory solution rather than a uniquely reproducible partition.

### Conclusion

This study demonstrates the value of integrating structured symptom extraction with narrative-based topic modeling to characterize cancer symptom experience in patient-generated social media narratives. Structured symptom extraction identified the occurrence and co-occurrence of predefined SPPADE symptoms, while narrative analysis contextualized these symptoms within broader experiences involving treatment decision-making, health care interaction, family responsibility, financial burden, and existential distress. Together, these complementary approaches provide a broader view of symptom experiences than either structured symptom assessment or topic-based analysis alone.

The findings also highlight the potential of patient-generated narratives as a complementary source of information for supportive cancer care. Although medication-based coping was more prominently represented than nonpharmacological strategies in the online narratives, this pattern should be understood as a feature of narrative representation rather than evidence of differences in actual use. Incorporating patient-generated narratives alongside structured symptom assessment may help identify aspects of symptom experience and supportive care that are less visible in conventional assessment, while their clinical utility warrants further evaluation.

## Supplementary material

10.2196/102862 Multimedia Appendix 1 Data collection and search strategy.

10.2196/102862 Multimedia Appendix 2 Large language model annotation prompts.

10.2196/102862 Multimedia Appendix 3 Cancer types and clinical staging.

10.2196/102862 Multimedia Appendix 4 BERTopic model selection grid search results.

10.2196/102862 Multimedia Appendix 5 Correspondence between the stable 5-dimensional solution and the original 13-topic solution.

10.2196/102862 Multimedia Appendix 6 Topics and representative patient quotes (n=19,392).

10.2196/102862 Multimedia Appendix 7 Topic-symptom co-occurrence matrix: prevalence of symptom dimensions across the 13 topics.

## Abbreviations

ARI: adjusted Rand index
c-TF-IDF: class-based term frequency–inverse document frequency
EOM: excess-of-mass
ePROM: electronic patient-reported outcome measure
HDBSCAN: Hierarchical Density-Based Spatial Clustering of Applications with Noise
LLM: large language model
NMI: normalized mutual information
NPV: negative predictive value
NRS: Numeric Rating Scale
PPV: positive predictive value
PRO: patient-reported outcome
PROM: patient-reported outcome measure
SPPADE: sleep disturbance, pain, physical function impairment, anxiety, depression, and low energy or fatigue
UMAP: Uniform Manifold Approximation and Projection
