# Supplementary material for: Characterizing Cancer Symptom Experiences and Related Psychosocial Concerns Through Chinese Social Media Narratives: Computational Text Analysis
Source: J Med Internet Res. 2026 Oct 20;28:e102862. doi: 10.2196/102862 (PMC13683996; doi:10.2196/102862)
Supplement: Supplementary file 1 [file jmir-v28-e102862-s001.docx]

**Multimedia Appendix 1. Data Collection and Search Strategy.**

**Search Terms**

Posts were retrieved using 33 Chinese search terms covering six SPPADE symptom domains and broader patient experience expressions. The same search terms were applied to both platforms. Terms are listed below by category.

| **Category** | **Search Terms (Chinese)** |
| --- | --- |
| Sleep Disturbance | 癌症失眠; 肿瘤睡不着; 癌症通宵没睡 |
| Pain | 癌症疼痛; 肿瘤癌痛; 癌症止痛 |
| Physical Function | 癌症虚弱; 肿瘤没力气; 癌症行动困难 |
| Anxiety | 癌症焦虑; 肿瘤恐惧; 癌症担心复发; 癌症担心病情 |
| Depression | 癌症抑郁; 肿瘤情绪低调; 癌症不想活; 肿瘤绝望 |
| Energy/Fatigue | 癌症疲乏; 肿瘤没精神; 癌症累得不行; 癌症非常虚 |
| Symptom Cluster | 癌症睡不着*疼*没力气*焦虑*抑郁 |
| Emotional Expressions | 癌症好难受; 癌症撑不下去; 癌症在线求助 |
| Daily Life Impact | 癌症生活质量差; 癌症做不了事; 肿瘤影响生活 |
| Patient Experience | 癌症真实感受; 肿瘤症状描述; 癌症心路历程; 癌症日常分享; 癌症个人体验 |

**Data Collection**

Data were collected from Xiaohongshu and Zhihu between November 2013 and February 2026. Data were collected programmatically from publicly accessible posts on both platforms using automated retrieval methods. Only publicly visible content was collected; no login credentials or private data were accessed. The initial corpus comprised 417,198 posts (Xiaohongshu: 375,658; Zhihu: 41,540).

**Deduplication**

Exact-match deduplication was applied at the post-content level, whereby posts sharing identical textual content were reduced to a single instance regardless of platform of origin or posting date. This procedure reduced the corpus to 338,913 unique posts.

**Inclusion and Exclusion Criteria**

Post eligibility was subsequently determined through a two-stage LLM-based cascade (see main text Methods). The applied criteria were as follows:

|  | **Criteria** |
| --- | --- |
| **Inclusion** | Written in Chinese; posted on Xiaohongshu or Zhihu within the specified collection period; first-person narrative in which the author explicitly describes their own cancer diagnosis, treatment, symptoms, or illness experience |
| **Exclusion** | Caregiver or family member accounts describing another person's experience; health professional advice, educational content, or scientific information; promotional or advertising content; expressions of support, encouragement, or well-wishes without personal illness narrative; posts for which authorship could not be determined |
