# Supplementary material for: Characterizing Cancer Symptom Experiences and Related Psychosocial Concerns Through Chinese Social Media Narratives: Computational Text Analysis
Source: J Med Internet Res. 2026 Oct 20;28:e102862. doi: 10.2196/102862 (PMC13683996; doi:10.2196/102862)
Supplement: Supplementary file 2 [file jmir-v28-e102862-s002.docx]

**Multimedia Appendix 2. LLM Annotation Prompts.**

GPT-4o-mini (OpenAI) was used for all annotation tasks. Four task-specific prompts were developed to structure the annotation of the social media corpus. All prompts were written in Chinese to match the language of the source data.

**Prompt 1: First-Person Patient Narrative Identification (Recall-Oriented Screening)**

Applied in the first round of a two-stage cascade to the full deduplicated corpus (n = 338,913 posts). Posts flagged as eligible (output = 1) were subsequently re-screened in a second precision-oriented round to verify genuine first-person patient narratives and exclude caregiver accounts, health information posts, and non-experiential content. The two-stage process yielded a final analytical corpus of 26,086 posts.

输入是一段癌症相关评论。请做二分类：是否为“患者本人自述”。
输出 1：作者明确是患者本人，并在第一人称叙述自己的诊断/治疗/检查/病程/症状/复发/生存等。
输出 0：作者不是患者本人，或仅为家属代述/转述/科普建议/讨论观点/祝福/广告/不确定。
仅输出 1 或 0，不输出其他内容。

**Prompt 2: SPPADE Symptom Extraction**

Applied to the final analytical corpus (n = 26,086 posts). Multi-label classification: a post may be assigned to multiple SPPADE dimensions simultaneously or to none. LLM performance was validated against independent human coding on a random sample (n = 300 posts); mean Cohen's κ = 0.714 (Rater A) and 0.754 (Rater B) across the 6 symptom domains.

你是一个医学文本信息抽取助手。你的任务是从患者自述文本中识别其是否提到了以下 SPPADE 症状维度中的一个或多个：
1. Sleep disturbance（睡眠障碍）2. Pain（疼痛）3. Physical function impairment（躯体功能受损/身体功能下降）4. Anxiety（焦虑）5. Depression（抑郁）6. Low energy / Fatigue（精力不足/疲乏）
这是一个多标签识别任务：一段文本中可以同时命中多个维度，也可以一个都不命中。
【标签定义】

* Sleep disturbance：包括失眠、入睡困难、早醒、睡眠浅、多梦、睡不着、睡眠质量差、嗜睡、睡眠紊乱等。* Pain：包括身体任何部位的疼痛、不适、刺痛、胀痛、隐痛、剧痛、酸痛、疼得厉害等。* Physical function impairment：包括行动受限、走路困难、起床困难、无法久站、无力完成日常活动、活动能力下降、自理能力下降、身体机能明显变差等。* Anxiety：包括紧张、焦虑、担心、害怕、恐慌、坐立不安、总是忧心、对病情过度担忧等。* Depression：包括情绪低落、沮丧、绝望、想哭、兴趣下降、消极、无助感、失去生活希望等。* Low energy / Fatigue：包括乏力、疲劳、没精神、很累、容易疲倦、精力差、虚弱、提不起劲等。
【识别原则】1. 只依据文本内容判断，不要添加文本中没有的信息。2. 若文本明确表达某症状当前存在，则标记为命中。3. 若文本明确否认某症状，则该标签不命中。4. 若文本同时提到过去和现在，以当前状态为准；若已明确缓解、消失，则不命中。5. 若只是可能、怀疑、转述他人意见，且患者本人未明确表达，则默认不命中。6. 同一句可能同时对应多个标签，需分别判断。7. 如果表达过于模糊，无法支持明确判断，则不要强行标注。8. 不要把一般性的疾病描述自动等同于 SPPADE 症状。
【输出要求】你必须且只能输出固定7行，严格按照以下顺序和格式，不得增加任何其他内容，不得加标题、编号、解释、引号或 Markdown：Sleep disturbance：<原文证据短语或 -1>，Pain：<原文证据短语或 -1>，Physical function impairment：<原文证据短语或 -1>，Anxiety：<原文证据短语或 -1>，Depression：<原文证据短语或 -1>
Low energy / Fatigue：<原文证据短语或 -1>，说明：<简要说明整体判断；若有否定、缓解、模糊表达，在此说明>
【证据提取要求】* 这里输出的不是概括总结，而是最能直接支持判断的“原文证据短语”。* 证据短语必须直接摘自原文，不得改写。* 多个证据短语之间用“&”连接。* 若该标签不命中，输出 -1。

**Prompt 3: Cancer Type and Disease Stage Extraction**

Applied to the final analytical corpus (n = 26,086 posts). Extraction is based solely on explicit textual evidence; inference from symptoms, treatments, or metastasis patterns is not permitted. Posts in which cancer type or stage could not be identified were coded as “not described.”

你是一个医学文本信息抽取助手。你的任务是从患者自述文本中抽取两类信息，并进行标准化输出：A. 癌症类型（只能选择一个）B. 分期/分型/分级（只能选择一个）
【总体要求】1. 只依据原文中明确表达的信息进行判断，不得根据症状、治疗方式、转移、复发、扩散等信息进行推断。2. 如果原文没有明确提到癌症类型，则“癌症类型”输出“未描述”。3. 如果原文没有明确提到分期/分型/分级信息，则“分期”输出“未描述”。4. 每一类只能输出一个结果；若原文中出现多个结果且无法判断哪个与患者本人最直接相关、表述最明确，则输出“未描述”。5. 说明部分只需简要说明判断依据；若输出“未描述”，需简要说明是“原文未明确提及”或“存在冲突，无法判断”。6. 不得输出任何额外内容，不得加标题、编号、示例、Markdown、引号或解释性扩展。
【A. 癌症类型可选项（只能选择一个）】
- 肺癌- 小细胞型肺癌- 非小细胞型肺癌- 鼻咽癌- 喉癌- 口腔癌- 食管癌- 胃癌- 十二指肠癌- 肝癌- 胆囊癌- 胆管癌- 胰腺癌- 结直肠癌- 肛门癌- 肾癌- 前列腺癌- 膀胱癌- 乳腺癌- 宫颈癌- 子宫内膜癌- 卵巢癌- 绒毛膜癌- 阴道癌- 白血病- 急性淋巴细胞白血病- 非急性淋巴细胞白血病- 慢性粒细胞性白血病- 霍奇金淋巴瘤- 非霍奇金淋巴瘤- 多发性骨髓瘤- 甲状腺癌- 垂体瘤- 脑胶质瘤- 低级别脑胶质瘤- 室管膜肿瘤- 间变性胶质瘤- 胶质母细胞瘤- 骨肉瘤- 黑色素瘤- 未描述
【B. 分期/分型/分级标准化输出（只能选择一个）】
1. TNM 临床分期：
- I期- II期- III期- IV期- 晚期
对应规则：- “1期/Ⅰ期/I期”统一输出为“I期”- “2期/Ⅱ期/II期”统一输出为“II期”- “3期/Ⅲ期/III期”统一输出为“III期”- “4期/Ⅳ期/IV期”统一输出为“IV期”- 原文明确写“晚期”但未给出具体期别，输出“晚期”
2. 小细胞型肺癌（SCLC）二期法：
- 局限期- 广泛期
3. 肝癌 CNLC：
- CNLC Ia- CNLC Ib- CNLC IIa- CNLC IIb- CNLC IIIa- CNLC IIIb- CNLC IV
4. 妇科 FIGO（宫颈癌、子宫内膜癌、卵巢癌）：
- FIGO I期- FIGO II期- FIGO III期- FIGO IV期
5. 淋巴瘤 Lugano（霍奇金淋巴瘤、非霍奇金淋巴瘤）：
- Lugano I期- Lugano II期- Lugano III期- Lugano IV期
6. 多发性骨髓瘤：
- I期- II期- III期- R2-ISS I期- R2-ISS II期- R2-ISS III期- R2-ISS IV期
说明：- 只有当原文明确出现 DS、ISS 或 R2-ISS 等分期体系信息时，才可按对应体系输出。- 若原文只写“骨髓瘤X期”但没有明确体系，可按“I期/II期/III期”输出。
7. 白血病相关分型：
- L1- L2- L3- M0- M1- M2- M3- M4- M5- M6- M7- 慢性期- 加速期- 急变期
说明：- 仅当原文明确出现这些分型/分期时才能输出，否则输出“未描述”。
8. 神经系统肿瘤分级：
- WHO 1级- WHO 2级- WHO 3级- WHO 4级
说明：- 仅当原文明确出现 WHO 分级、1级、2级、3级、4级等信息时才能输出，否则输出“未描述”。
9. 若原文未出现任何可标准化的分期/分型/分级信息：
- 未描述
【特别注意】1. “转移”“复发”“扩散”本身不等同于 IV期 或 晚期，只有原文明确出现“IV期/4期/Ⅳ期/晚期”时，才可输出相关结果。2. 不得根据癌症类型去推测其常见分期体系；必须以原文明确提到的信息为准。3. 不得根据药物、手术、放疗、化疗、靶向治疗、免疫治疗等反推分期。4. 若文本中同时出现既往与当前多个不同说法，且无法明确哪个才是患者本人的最终信息，则输出“未描述”。
【输出要求（必须且只能输出 3 行，不得增加任何其他内容）】
癌症类型：<标准化类别>分期：<标准化类别>说明：<简要说明判断依据>

**Prompt 4: Coping Strategy Extraction**

Applied to posts with at least one SPPADE symptom identified. Input includes both the post text and the list of SPPADE dimensions already identified for that post (from Prompt 2). Extracted strategies were subsequently standardised into 10 categories through iterative review.

请根据下面提供的“患者自述文本”和“SPPADE症状列表”，识别该患者在各个 SPPADE 症状维度下体现出的“症状应对方式/真实需求”。
【任务说明】你不需要再次判断症状是否存在。SPPADE症状列表已经给出，你只需要基于：1. 患者自述文本2. 已识别出的 SPPADE 症状列表来分析每个症状维度是否体现了相应的应对方式或真实需求。SPPADE 六个维度为：- Sleep disturbance- Pain- Physical function impairment- Anxiety- Depression- Low energy / Fatigue
【应对方式/真实需求的范围】包括但不限于以下类型：
- 药物应对：止痛药、安眠药、抗焦虑药、抗抑郁药、中药、营养补充剂等
- 医疗支持：咨询医生、复诊就医、住院、治疗调整、心理门诊、康复训练等
- 心理支持：心理疏导、情绪安慰、倾诉、家人支持、病友互助等
- 生活方式调节：休息、锻炼、饮食调节、作息调整、放松训练等
- 社会支持：家属照顾、朋友帮助、病友群、线上求助等
- 未采取措施：没有处理、没吃药、不知道怎么办、只能忍着、硬扛等
【判断规则】1. 只依据原文明确表达的信息判断，不要补充推断。2. 必须结合给定的 SPPADE 症状列表进行分析：	- 若某个维度不在给定的 SPPADE 症状列表中，则该维度输出 -1。	- 若某个维度在给定的 SPPADE 症状列表中，但原文没有体现明确的应对方式或真实需求，也输出 -1。3. 应对方式必须与该症状维度直接相关，不能把针对其他症状的应对方式错误归到当前维度。4. 若原文表达的是“想问怎么办”“希望有人建议”“有没有人有经验”“想找人聊聊”等，这也属于真实需求，可以提取出来。5. 若原文明确表示“没有采取措施”“没办法”“只能忍着”“没吃药”“没处理”，可归为“未采取措施”。6. 同一维度下的多个应对方式用“&”连接。7. 输出尽量使用简洁短语，不要输出完整长句。8. 不要把泛泛而谈的情绪表达误当成应对方式；只有涉及处理方法、支持需求、求助行为或未采取措施时，才算应对方式/真实需求。
【输出要求】必须且只能输出固定7行，严格按照以下顺序和格式，不得增加任何其他内容，不得加标题、编号、解释、引号或 Markdown：
Sleep disturbance：<应对方式 或 -1>Pain：<应对方式 或 -1>Physical function impairment：<应对方式 或 -1>Anxiety：<应对方式 或 -1>Depression：<应对方式 或 -1>Low energy / Fatigue：<应对方式 或 -1>说明：<简要说明哪些症状提到了应对方式；若都没有，则说明原文未体现明确应对方式>
