# Supplementary material for: Characterizing Cancer Symptom Experiences and Related Psychosocial Concerns Through Chinese Social Media Narratives: Computational Text Analysis
Source: J Med Internet Res. 2026 Oct 20;28:e102862. doi: 10.2196/102862 (PMC13683996; doi:10.2196/102862)
Supplement: Supplementary file 3 [file jmir-v28-e102862-s003.docx]

**Multimedia Appendix 3. Cancer Types and Clinical Staging.**

| **System** | **Cancer Type** | **Clinical Staging**  **(Default:TNM Staging^a^)** |
| --- | --- | --- |
| Respiratory System | Lung Cancer | Stage Ⅰ–Ⅳ |
|  | Small Cell Lung Cancer(SCLC) | Limited Stage,Extensive Stage^b^ |
|  | Non-Small Cell Lung Cancer(NSCLC) | Stage Ⅰ–Ⅳ |
|  | Nasopharyngeal Carcinoma | Stage Ⅰ–Ⅳ |
|  | Laryngeal Cancer | Stage Ⅰ–Ⅳ |
| Digestive System | Oral Cancer | Stage Ⅰ–Ⅳ |
|  | Esophageal Cancer | Stage Ⅰ–Ⅳ |
|  | Gastric Cancer | Stage Ⅰ–Ⅳ |
|  | Duodenal Cancer | Stage Ⅰ–Ⅳ |
|  | Liver Cancer | Stage Ⅰ–Ⅳ(CNLC Staging^c^) |
|  | Gallbladder Cancer | Stage Ⅰ–Ⅳ |
|  | Cholangiocarcinoma | Stage Ⅰ–Ⅳ |
|  | Pancreatic Cancer | Stage Ⅰ–Ⅳ |
|  | Colorectal Cancer | Stage Ⅰ–Ⅳ |
|  | Anal Cancer | Stage Ⅰ–Ⅳ |
| Urinary and Male Genital System | Renal Cancer | Stage Ⅰ–Ⅳ |
|  | Prostate Cancer | Stage Ⅰ–Ⅳ |
|  | Bladder Cancer | Stage Ⅰ–Ⅳ |
| Gynecologic and Breast Disorders | Breast Cancer | Stage Ⅰ–Ⅳ |
|  | Cervical Cancer | Stage Ⅰ–Ⅳ (FIGO Staging^d^) |
|  | Endometrial Cancer | Stage Ⅰ–Ⅳ (FIGO Staging) |
|  | Ovarian Cancer | Stage Ⅰ–Ⅳ (FIGO Staging) |
|  | Choriocarcinoma | Stage Ⅰ–Ⅳ |
|  | Vaginal Cancer | Stage Ⅰ–Ⅳ |
| Hematologic and Lymphatic System | Leukemia | No Clinical Staging |
|  | Acute Lymphoblastic Leukemia  (ALL) | L1–L3 Subtypes  (FAB Classification^e^) |
|  | Non-Acute Lymphoblastic Leukemia | M0–M7 Subtypes  (FAB Classification) |
|  | Chronic Myeloid Leukemia  (CML) | Chronic Phase, Accelerated Phase, Blast Phase  (WHO Criteriae) |
|  | Hodgkin Lymphoma(HL) | Stage Ⅰ–Ⅳ(Lugano Staging^f^) |
|  | Non-Hodgkin Lymphoma  (NHL) | Stage Ⅰ–Ⅳ (Lugano Staging) |
|  | Multiple Myeloma | Stage Ⅰ–Ⅲ (DS/ISS Staging^g^);  Stage Ⅰ–Ⅳ (R2-ISS Staging^g^) |
| Endocrine System | Thyroid Cancer | Stage Ⅰ–Ⅳ |
|  | Pituitary Tumor | No Staging^i^ |
| Nervous System | Glioma | No Staging,Grades 1–4 per WHO Criteria^h^ |
|  | Low-Grade Glioma (Diffuse Astrocytoma, Oligodendroglioma) | Grade 1 |
|  | Ependymal Tumors | Grade 2–3 |
|  | Anaplastic Glioma | Grade 3 |
|  | Glioblastoma | Grade 4 |
| Skeletal and Soft Tissue | Osteosarcoma | Stage Ⅰ–Ⅲ |
|  | Melanoma | Stage Ⅰ–Ⅳ |

* **a:**The American Joint Committee on Cancer (AJCC) and the International Union Against Cancer (UICC) have established international tumor staging criteria.This system classifies tumor stages based on three dimensions:T (primary tumor extent),N (regional lymph node metastasis),and M (distant metastasis).The combined indicators correspond to clinical stages I to IV.

**b:**The National Comprehensive Cancer Network (NCCN) panel recommends combining the AJCC TNM staging system with the VALG two-stage classification for small cell lung cancer,which can better guide clinical treatment and evaluate prognosis.

**c:**Liver cancer staging is critical for therapeutic regimen selection and prognostic assessment.Multiple international staging systems are available,including the Barcelona Clinic Liver Cancer (BCLC) staging,TNM staging,staging criteria formulated by the Japanese Society of Hepatology and the Asian Pacific Association for the Study of the Liver.In accordance with national conditions and clinical experience in China, the China Liver Cancer Staging (CNLC) system has been established based on patients’ performance status (PS),hepatic tumor lesions and liver function.It comprises CNLC stage Ia, Ib, IIa, IIb, IIIa, IIIb and IV.

**d:**The International Federation of Gynecology and Obstetrics (FIGO) staging is a standardized classification system formulated for gynecological malignant tumors such as cervical cancer and endometrial carcinoma.

**e:**Conventional staging is generally not applied to acute leukemia due to its rapid progression,and comprehensive therapy serves as the core intervention.Diagnosis and treatment guidance rely mainly on cell classification and genetic characteristics for typing.In contrast,chronic leukemia is clearly staged in accordance with WHO criteria based on disease progression.

**f:**The Lugano classification for lymphoma is a standardized system for staging and therapeutic efficacy evaluation of Hodgkin lymphoma and non-Hodgkin lymphoma. It determines lesion scope, guides treatment and judges prognosis according to imaging findings including PET-CT and clinical manifestations.

**g:**The Durie-Salmon (DS) staging system stratifies multiple myeloma into stage I, II and III according to hemoglobin level,blood calcium concentration,bone lesions and M protein content.The International Staging System (ISS) assesses prognosis and categorizes patients into three stages based on serum albumin and β2-microglobulin levels.The revised second-version International Staging System (R2-ISS) divides the disease into stage I to IV.Based on ISS,the Revised International Staging System (R-ISS) incorporates lactate dehydrogenase level and cytogenetic factors,enabling more objective and accurate prognostic evaluation.

**h:**Widely adopted in most malignancies,the traditional TNM staging system is not applicable to glioblastoma due to its unique growth pattern and biological behavior.Tumor cells infiltrate adjacent normal brain tissues with irregular boundaries,rendering tumor size and lymph node metastasis indicators in TNM staging clinically insignificant.

**i:**Tumor severity and dissemination degree are generally described by staging.No unified standard staging system exists for pituitary adenoma. Relevant examinations will be conducted upon diagnosis to identify tumor invasion into intracranial tissues or other systemic organs.
