# Supplementary material for: Characterizing Cancer Symptom Experiences and Related Psychosocial Concerns Through Chinese Social Media Narratives: Computational Text Analysis
Source: J Med Internet Res. 2026 Oct 20;28:e102862. doi: 10.2196/102862 (PMC13683996; doi:10.2196/102862)
Supplement: Supplementary file 4 [file jmir-v28-e102862-s004.docx]

**Multimedia Appendix 4. BERTopic Model Selection Grid Search Results.**

**Table A. The 18 BERTopic configurations evaluated during primary model selection, ranked by topic coherence score (Cᵥ, descending).**

| **Rank** | **n_components** | **n_neighbors** | **Min cluster size** | **No. of topics** | **Coherence score (Cᵥ)** |
| --- | --- | --- | --- | --- | --- |
| 1 | 10 | 15 | 125 | 2 | 0.5829 |
| **2** | **2** | **10** | **100** | **14** | **0.5799** |
| 3 | 14 | 10 | 100 | 18 | 0.5740 |
| 4 | 6 | 5 | 100 | 5 | 0.5637 |
| 5 | 2 | 5 | 150 | 15 | 0.5624 |
| 6 | 6 | 5 | 90 | 19 | 0.5609 |
| 7 | 6 | 10 | 75 | 21 | 0.5602 |
| 8 | 10 | 10 | 90 | 17 | 0.5593 |
| 9 | 14 | 5 | 90 | 23 | 0.5591 |
| 10 | 2 | 10 | 90 | 22 | 0.5581 |
| 11 | 2 | 10 | 75 | 26 | 0.5564 |
| 12 | 2 | 15 | 100 | 7 | 0.5526 |
| 13 | 2 | 10 | 150 | 6 | 0.5512 |
| 14 | 10 | 5 | 125 | 4 | 0.5486 |
| 15 | 10 | 5 | 100 | 20 | 0.5457 |
| 16 | 2 | 15 | 75 | 16 | 0.5366 |
| 17 | 2 | 20 | 50 | 10 | 0.5283 |
| 18 | 2 | 5 | 100 | 3 | 0.5068 |

* The 18 configurations evaluated during the primary model selection (see Methods, "Topic Model Optimization"; Results, "Model Selection"). The originally selected model (n_components = 2, n_neighbors = 10, min_cluster_size = 100) is shown in bold. The original analysis selected this configuration based on a combination of semantic coherence and topic interpretability. The coherence-top-ranked configuration (n_components = 10, n_neighbors = 15, min_cluster_size = 125) yielded only 2 topics and was not substantively interpretable.

**Table B. All 112 BERTopic grid-search configurations, ranked by topic coherence score (Cᵥ, descending).**

| **Rank** | **n_components** | **n_neighbors** | **Min cluster size** | **No. of topics** | **Coherence score (Cᵥ)** |
| --- | --- | --- | --- | --- | --- |
| 1 | 10 | 15 | 125 | 2 | 0.5829 |
| 2 | 14 | 15 | 100 | 2 | 0.5828 |
| 3 | 6 | 15 | 100 | 2 | 0.5828 |
| 4 | 6 | 20 | 100 | 2 | 0.5828 |
| 5 | 10 | 20 | 100 | 2 | 0.5828 |
| 6 | 2 | 15 | 150 | 2 | 0.5828 |
| 7 | 6 | 15 | 150 | 2 | 0.5828 |
| 8 | 2 | 20 | 100 | 2 | 0.5828 |
| 9 | 2 | 20 | 150 | 2 | 0.5828 |
| 10 | 6 | 20 | 125 | 2 | 0.5828 |
| 11 | 14 | 20 | 125 | 2 | 0.5828 |
| 12 | 10 | 20 | 125 | 2 | 0.5828 |
| 13 | 2 | 15 | 125 | 2 | 0.5828 |
| 14 | 6 | 15 | 125 | 2 | 0.5828 |
| 15 | 2 | 20 | 125 | 2 | 0.5828 |
| 16 | 6 | 20 | 150 | 2 | 0.5828 |
| 17 | 2 | 20 | 75 | 2 | 0.5828 |
| 18 | 6 | 15 | 75 | 2 | 0.5828 |
| 19 | 14 | 15 | 75 | 2 | 0.5828 |
| 20 | 14 | 15 | 90 | 2 | 0.5828 |
| 21 | 6 | 20 | 90 | 2 | 0.5828 |
| 22 | 6 | 15 | 90 | 2 | 0.5828 |
| 23 | 2 | 15 | 90 | 2 | 0.5828 |
| 24 | 2 | 20 | 90 | 2 | 0.5828 |
| 25 | 10 | 20 | 90 | 2 | 0.5828 |
| **26** | **2** | **10** | **100** | **14** | **0.5799** |
| 27 | 14 | 10 | 100 | 18 | 0.5740 |
| 28 | 6 | 10 | 100 | 18 | 0.5736 |
| 29 | 14 | 10 | 90 | 18 | 0.5680 |
| 30 | 2 | 5 | 125 | 18 | 0.5679 |
| 31 | 6 | 10 | 90 | 18 | 0.5650 |
| 32 | 10 | 10 | 100 | 18 | 0.5642 |
| 33 | 6 | 5 | 100 | 5 | 0.5637 |
| 34 | 2 | 5 | 150 | 15 | 0.5624 |
| 35 | 6 | 5 | 90 | 19 | 0.5609 |
| 36 | 6 | 10 | 75 | 21 | 0.5602 |
| 37 | 10 | 10 | 90 | 17 | 0.5593 |
| 38 | 14 | 5 | 90 | 23 | 0.5591 |
| 39 | 2 | 10 | 90 | 22 | 0.5581 |
| 40 | 2 | 10 | 125 | 14 | 0.5577 |
| 41 | 14 | 10 | 75 | 22 | 0.5568 |
| 42 | 2 | 10 | 75 | 26 | 0.5564 |
| 43 | 10 | 10 | 75 | 22 | 0.5550 |
| 44 | 2 | 15 | 100 | 7 | 0.5526 |
| 45 | 2 | 10 | 150 | 6 | 0.5512 |
| 46 | 10 | 5 | 125 | 4 | 0.5486 |
| 47 | 10 | 5 | 150 | 4 | 0.5466 |
| 48 | 10 | 5 | 100 | 20 | 0.5457 |
| 49 | 10 | 5 | 90 | 22 | 0.5440 |
| 50 | 6 | 20 | 75 | 21 | 0.5411 |
| 51 | 6 | 5 | 75 | 26 | 0.5390 |
| 52 | 10 | 15 | 75 | 22 | 0.5369 |
| 53 | 2 | 15 | 75 | 16 | 0.5366 |
| 54 | 14 | 5 | 50 | 26 | 0.5364 |
| 55 | 6 | 5 | 150 | 6 | 0.5361 |
| 56 | 10 | 20 | 150 | 5 | 0.5354 |
| 57 | 14 | 5 | 25 | 26 | 0.5341 |
| 58 | 6 | 5 | 50 | 26 | 0.5339 |
| 59 | 10 | 15 | 25 | 26 | 0.5338 |
| 60 | 10 | 15 | 150 | 5 | 0.5337 |
| 61 | 6 | 20 | 50 | 26 | 0.5336 |
| 62 | 14 | 5 | 75 | 26 | 0.5330 |
| 63 | 10 | 5 | 50 | 26 | 0.5328 |
| 64 | 10 | 10 | 125 | 5 | 0.5324 |
| 65 | 6 | 10 | 150 | 5 | 0.5323 |
| 66 | 10 | 5 | 75 | 26 | 0.5322 |
| 67 | 2 | 15 | 50 | 26 | 0.5321 |
| 68 | 10 | 5 | 25 | 26 | 0.5320 |
| 69 | 2 | 15 | 25 | 26 | 0.5318 |
| 70 | 2 | 5 | 25 | 26 | 0.5307 |
| 71 | 10 | 20 | 50 | 26 | 0.5305 |
| 72 | 14 | 20 | 150 | 5 | 0.5301 |
| 73 | 14 | 20 | 75 | 7 | 0.5296 |
| 74 | 10 | 15 | 50 | 26 | 0.5295 |
| 75 | 2 | 20 | 50 | 10 | 0.5283 |
| 76 | 6 | 10 | 50 | 26 | 0.5271 |
| 77 | 6 | 5 | 125 | 4 | 0.5271 |
| 78 | 10 | 20 | 25 | 26 | 0.5265 |
| 79 | 2 | 10 | 50 | 26 | 0.5251 |
| 80 | 14 | 15 | 50 | 26 | 0.5251 |
| 81 | 14 | 15 | 25 | 26 | 0.5246 |
| 82 | 10 | 10 | 50 | 26 | 0.5228 |
| 83 | 6 | 5 | 25 | 26 | 0.5213 |
| 84 | 2 | 10 | 25 | 26 | 0.5195 |
| 85 | 10 | 20 | 75 | 4 | 0.5167 |
| 86 | 2 | 5 | 50 | 26 | 0.5154 |
| 87 | 14 | 5 | 150 | 4 | 0.5149 |
| 88 | 14 | 15 | 150 | 5 | 0.5133 |
| 89 | 2 | 5 | 75 | 4 | 0.5130 |
| 90 | 2 | 5 | 90 | 4 | 0.5130 |
| 91 | 14 | 5 | 100 | 5 | 0.5126 |
| 92 | 14 | 10 | 50 | 26 | 0.5124 |
| 93 | 6 | 20 | 25 | 26 | 0.5113 |
| 94 | 6 | 15 | 50 | 26 | 0.5104 |
| 95 | 2 | 20 | 25 | 26 | 0.5091 |
| 96 | 14 | 15 | 125 | 5 | 0.5070 |
| 97 | 14 | 20 | 50 | 7 | 0.5069 |
| 98 | 2 | 5 | 100 | 3 | 0.5068 |
| 99 | 14 | 20 | 25 | 26 | 0.5042 |
| 100 | 10 | 10 | 150 | 4 | 0.5010 |
| 101 | 10 | 10 | 25 | 26 | 0.4998 |
| 102 | 14 | 10 | 25 | 26 | 0.4998 |
| 103 | 6 | 10 | 25 | 26 | 0.4975 |
| 104 | 6 | 15 | 25 | 26 | 0.4964 |
| 105 | 14 | 5 | 125 | 4 | 0.4887 |
| 106 | 14 | 10 | 125 | 4 | 0.4812 |
| 107 | 10 | 15 | 90 | 4 | 0.4778 |
| 108 | 10 | 15 | 100 | 4 | 0.4744 |
| 109 | 14 | 10 | 150 | 4 | 0.4744 |
| 110 | 6 | 10 | 125 | 4 | 0.4744 |
| 111 | 14 | 20 | 90 | 4 | 0.4680 |
| 112 | 14 | 20 | 100 | 4 | 0.4637 |

* All 112 configurations of the expanded grid search are reported. The 18 primary model selection configurations (Table A) are a subset of this grid, ranked by topic coherence score (Cᵥ, descending). The originally selected model (n_components = 2, n_neighbors = 10, min_cluster_size = 100) is shown in bold; the original analysis selected this configuration based on a combination of semantic coherence and topic interpretability (see Methods). The coherence-top-ranked configuration (n_components = 10, n_neighbors = 15, min_cluster_size = 125) yielded only 2 topics and was not substantively interpretable. Subsequent stability assessment (Tables C and D) demonstrated that 2-dimensional UMAP configurations were highly unstable across random seeds; however, a stable 5-dimensional alternative configuration (n_components = 5, n_neighbors = 10, min_cluster_size = 75), which falls outside this original coherence-ranked grid, showed high correspondence with the original 13-topic structure and is reported as robustness evidence.

**Table C. Stability screen of 12 candidate BERTopic configurations (3 random seeds each).**

| **n_components** | **n_neighbors** | **Min cluster size** | **No. of topics (range)** | **Cᵥ (mean)** | **Median pairwise ARI** | **Mean keyword overlap** | **Max ARI vs original 13-topic solution** |
| --- | --- | --- | --- | --- | --- | --- | --- |
| 10 | 15 | 100 | 2–2 | 0.5828 | 0.9944 | 1.000 | 0.026 |
| 10 | 10 | 75 | 19–23 | 0.5599 | 0.8487 | 0.826 | 0.806 |
| 5 | 10 | 75 | 19–23 | 0.5554 | 0.8451 | 0.827 | 0.808 |
| 2 | 15 | 75 | 4–25 | 0.5432 | 0.1070 | 0.517 | 0.602 |
| 2 | 10 | 100 | 2–7 | 0.5790 | 0.0746 | 0.679 | 0.487 |
| 2 | 15 | 100 | 2–7 | 0.5718 | 0.0728 | 0.709 | 0.506 |
| 2 | 10 | 75 | 2–22 | 0.5555 | 0.0602 | 0.596 | 0.102 |
| 10 | 10 | 100 | 3–20 | 0.5583 | 0.0317 | 0.567 | 0.813 |
| 10 | 15 | 75 | 3–22 | 0.5522 | 0.0294 | 0.579 | 0.783 |
| 5 | 15 | 100 | 2–14 | 0.5807 | 0.0234 | 0.619 | 0.854 |
| 5 | 10 | 100 | 2–20 | 0.5791 | 0.0216 | 0.545 | 0.818 |
| 5 | 15 | 75 | 2–19 | 0.5734 | 0.0209 | 0.593 | 0.800 |

* Configurations are ranked by median pairwise adjusted Rand index (ARI) across the 3 seeds. Two-dimensional configurations (n_components = 2) showed near-zero cross-seed agreement (median pairwise ARI 0.06–0.11), whereas the n_neighbors = 10, min_cluster_size = 75 family achieved high cross-seed agreement. The configuration with the highest pairwise ARI (n_components = 10, n_neighbors = 15, min_cluster_size = 100) was stably degenerate, collapsing to 2 topics in every run, illustrating that stability alone does not certify validity. The stable alternative configuration (n_components = 5, n_neighbors = 10, min_cluster_size = 75) jointly satisfied the selection criteria: high cross-seed stability, a non-degenerate topic count consistent with the original solution, and high correspondence with the original 13-topic solution.

**Table D. Confirmatory multi-seed analysis of the stable alternative configuration (n_components = 5, n_neighbors = 10, min_cluster_size = 75; 11 random seeds).**

| **Random seed** | **No. of clusters** | **Outliers before reassignment, n (%)** | **Cᵥ** | **ARI vs original 13-topic solution** | **Median pairwise ARI vs other seeds** | **Note** |
| --- | --- | --- | --- | --- | --- | --- |
| 0 | 20 | 3,930 (20.3%) | 0.5545 | 0.798 | 0.946 |  |
| 1 | 23 | 4,036 (20.8%) | 0.5529 | 0.701 | 0.843 |  |
| 2 | 2 | 13 (0.1%) | 0.5829 | 0.026 | 0.021 | Degenerate solution (reported; excluded from representative selection) |
| 3 | 21 | 3,731 (19.2%) | 0.5569 | 0.806 | 0.948 |  |
| 4 | 18 | 4,119 (21.2%) | 0.5635 | 0.802 | 0.947 |  |
| 5 | 20 | 4,092 (21.1%) | 0.5563 | 0.808 | 0.934 |  |
| 6 | 3 | 105 (0.5%) | 0.5252 | 0.039 | 0.031 | Degenerate solution (reported; excluded from representative selection) |
| 7 | 20 | 4,237 (21.8%) | 0.5526 | 0.806 | 0.948 |  |
| 8 | 20 | 3,984 (20.5%) | 0.5499 | 0.808 | 0.951 | Medoid solution — selected as the final representative |
| 9 | 20 | 4,180 (21.6%) | 0.5608 | 0.805 | 0.945 |  |
| 42 | 19 | 4,029 (20.8%) | 0.5590 | 0.808 | 0.951 |  |

* A solution was classified as degenerate when it collapsed to 3 or fewer clusters; this criterion was specified based on the configuration screen (Table C), in which no configuration produced a stable intermediate cluster count (4–17) across seeds, and was applied to the confirmatory analysis. All 11 solutions, including the two degenerate ones, are reported. Among the 9 non-degenerate solutions (18–23 clusters), the median pairwise ARI ranged from 0.843 to 0.951 and the median ARI against the original 13-topic solution was 0.806. The confirmatory solutions showed high correspondence with the original 13-topic solution (median ARI = 0.806), supporting the robustness of the major semantic structure. After embedding-based outlier reassignment (default similarity threshold of 0), all 19,392 posts were assigned to a cluster (noise = 0%).
