# Supplementary material for: Characterizing Cancer Symptom Experiences and Related Psychosocial Concerns Through Chinese Social Media Narratives: Computational Text Analysis
Source: J Med Internet Res. 2026 Oct 20;28:e102862. doi: 10.2196/102862 (PMC13683996; doi:10.2196/102862)
Supplement: Supplementary file 5 [file jmir-v28-e102862-s005.docx]

**Multimedia Appendix 5. Correspondence Between the Stable 5-Dimensional Solution and the Original 13-Topic Solution.**

A stable 5-dimensional BERTopic solution (n_components = 5, n_neighbors = 10, min_cluster_size = 75) was evaluated as a post hoc robustness check on the original 13-topic solution. This stable alternative comprised 20 fine-grained clusters (C0–C19). The correspondence between these 20 clusters and the original 13 topics (T0–T12) was assessed to determine whether the major semantic organisation of the original solution was preserved under a more stable configuration. The high correspondence (ARI = 0.808; weighted purity = 0.910) confirms that the major thematic structure is not an artefact of a single stochastic run. The original 13-topic solution was retained as the interpretive framework for the manuscript.

**Table A. The 20 clusters from the stable 5-dimensional solution.**

| **Cluster** | **n** | **Topkeywords(Chinese,c-TF-IDF)** | **Topkeywords(Englishgloss)** | **Assigneddomain** |
| --- | --- | --- | --- | --- |
| C0 | 489 | 家属; 原位; 医护人员; 癌症; 医院; 人生; 好转; 检查 | family member; in situ; healthcare provider; cancer; hospital; life; improvement; examination | T0 |
| C1 | 248 | 肝上; 肝转; 肝功能; 肝脏; 肝硬化; 介入; 转移; 乙肝 | suprahepatic; liver metastasis; liver function; liver; cirrhosis; interventional therapy; metastasis; hepatitis B | T1 |
| C2 | 149 | 费用; 医保; 进口; 医院; 一个月; 治疗; 用药; 手术 | cost; medical insurance; imported drugs; hospital; one month; treatment; medication; surgery | T3 |
| C3 | 7,127 | 化疗; 癌症; 手术; 免疫治疗; 进展期; 转移; 治疗; 靶向 | chemotherapy; cancer; surgery; immunotherapy; advanced stage; metastasis; treatment; targeted therapy | T4 |
| C4 | 137 | 明天; 检查; 增强ct; 出院; 核磁; 方案; 医院; 复查 | tomorrow; examination; contrast-enhanced CT; discharge; MRI; treatment plan; hospital; follow-up | T2 |
| C5 | 175 | 谢谢; 感谢; 合十; 拜托; 医护人员; 好转; 韧性; 玫瑰 | thank you; gratitude; folded hands; plea; healthcare provider; improvement; resilience; rose | T8 |
| C6 | 338 | 吗啡; 用药; 芬太尼; 止痛; 羟考酮; 止痛药; 作用; 副作用 | morphine; medication; fentanyl; pain relief; oxycodone; analgesics; effect; side effects | T7 |
| C7 | 865 | 睡眠; 昼夜感受; 睡觉; 不适; 疲乏; 半夜; 焦虑; 意识模糊 | sleep; day–night perception; sleeping; discomfort; fatigue; midnight; anxiety; confusion | T5 |
| C8 | 157 | 呼吸; 氧气; 呼吸困难; 血氧; 吸氧; 呼吸机; 血压; 喘气 | breathing; oxygen; dyspnoea; blood oxygen; oxygen inhalation; ventilator; blood pressure; gasping | T12 |
| C9 | 417 | 咳嗽; 发烧; 肺部; 肺炎; 喉咙; 症状; 检查; 结节 | cough; fever; pulmonary; pneumonia; throat; symptoms; examination; nodule | T12 |
| C10 | 2,200 | 焦虑; 家属; 抑郁; 不适; 人生; 外界支持; 孩子; 韧性 | anxiety; family member; depression; discomfort; life; external support; children; resilience | T6 |
| C11 | 212 | 意识模糊; 清醒; 说胡话; 不适; 怀疑; 癫痫; 状态; 转移 | confusion; lucidity; delirious speech; discomfort; suspicion; epilepsy; state; metastasis | T10 |
| C12 | 542 | 躯体部位; 腹水; 转移; 身体; 肚子; 发现; 淋巴结; 增强ct | body site; ascites; metastasis; body; abdomen; discovery; lymph node; contrast-enhanced CT | T11 |
| C13 | 979 | 食欲问题; 营养; 吃饭; 不适; 意识模糊; 腹水; 二便情况; 症状 | appetite problem; nutrition; eating; discomfort; confusion; ascites; bowel/bladder status; symptoms | T10 |
| C14 | 152 | 耐药; 副作用; 靶向; 用药; 作用; 单抗; 一年; 很快 | drug resistance; side effects; targeted therapy; medication; effect; monoclonal antibody; one year; rapid | T8 |
| C15 | 751 | 疼痛; 止痛; 止痛药; 不痛; 剧痛; 用药; 作用; 不适 | pain; pain relief; analgesics; pain-free; severe pain; medication; effect; discomfort | T8 |
| C16 | 1,184 | 人生; 死亡; 不适; 韧性; 求生欲; 希望; 外界支持; 意义 | life; death; discomfort; resilience; will to live; hope; external support; meaning | T9 |
| C17 | 485 | 治疗; 转移; 靶向; 用药; 方案; 放疗; 介入; 好转 | treatment; metastasis; targeted therapy; medication; regimen; radiotherapy; interventional therapy; improvement | T8 |
| C18 | 1,719 | 手术; 医护人员; 医院; 检查; 出院; 转移; 异地就医; 发现 | surgery; healthcare provider; hospital; examination; discharge; metastasis; out-of-town care; discovery | T8 |
| C19 | 1,066 | 一年; 两年; 一个月; 半年; 复发; 发现; 术后; 进展期 | one year; two years; one month; half a year; recurrence; discovery; postoperative; advanced stage | T8 |

**Table B. Correspondence between the stable 5-dimensional solution (20 clusters) and the original 13-topic solution.**

| **Thematic domain** | **Member clusters (n)** | **Domain n** | **Purity (share of domain posts from the corresponding original topic)** | **Coverage (share of original topic posts captured by the domain)** |
| --- | --- | --- | --- | --- |
| T0 Family Involvement and Care Decision-Making | C0 (489) | 489 | 0.992 | 0.951 |
| T1 Liver Metastasis and Hepatic Dysfunction | C1 (248) | 248 | 0.968 | 0.945 |
| T2 Diagnostic Process and Anticipatory Anxiety | C4 (137) | 137 | 0.876 | 0.727 |
| T3 Financial Burden and Access to Treatment | C2 (149) | 149 | 0.893 | 0.831 |
| T4 Cancer Treatment Trajectory and Therapeutic Strategies | C3 (7,127) | 7,127 | 0.984 | 0.974 |
| T5 Sleep Disturbance with Day–Night Variation | C7 (865) | 865 | 0.922 | 0.935 |
| T6 Psychological Distress: Anxiety and Depression | C10 (2,200) | 2,200 | 0.871 | 0.913 |
| T7 Opioid Use and Pain Management | C6 (338) | 338 | 0.828 | 0.778 |
| T8 Healthcare Interaction and Treatment Experience | C5 (175); C14 (152); C15 (751); C17 (485); C18 (1,719); C19 (1,066) | 4,348 | 0.856 | 0.907 |
| T9 Death-related Cognition and Existential Distress | C16 (1,184) | 1,184 | 0.904 | 0.796 |
| T10 Appetite Loss, Nutrition, and Physical Decline | C11 (212); C13 (979) | 1,191 | 0.884 | 0.772 |
| T11 Metastasis and Physical Manifestations | C12 (542) | 542 | 0.745 | 0.813 |
| T12 Dyspnea and Respiratory Support | C8 (157); C9 (417) | 574 | 0.706 | 0.837 |
| Overall (weighted by domain size) | — | — | 0.910 | — |

* Purity and coverage were computed from the full 13 × 20 cross-tabulation between the original 13-topic assignments and the 20 stable clusters (archived with the analysis code). Ten of the 13 topics correspond to a single stable cluster one-to-one; topics T8, T10, and T12 each comprise multiple fine-grained clusters, indicating that the corresponding original topics are further subdivided in the stable solution.
