# Supplementary material for: Characterizing Cancer Symptom Experiences and Related Psychosocial Concerns Through Chinese Social Media Narratives: Computational Text Analysis
Source: J Med Internet Res. 2026 Oct 20;28:e102862. doi: 10.2196/102862 (PMC13683996; doi:10.2196/102862)
Supplement: Supplementary file 6 [file jmir-v28-e102862-s006.docx]

**Multimedia Appendix 6. Topics and representative patient quotes (n = 19,392).**

| **Topic** | **Representative Quote** |
| --- | --- |
| T0 Family Involvement and Care Decision-Making | "Late-stage cancer is a bottomless pit. Nobody knows how much it will cost or whether it can be treated. My family doesn't have that kind of money or energy." （原文：癌症晚期本来就是一个无底洞，谁也不知道到底要花多少钱，到底能不能治好，我家没有这么多金钱和精力。） |
| T1 Liver Metastasis and Hepatic Dysfunction | "Jaundice. Bilirubin over 500. Multiple liver abscesses, the drainage isn't working. I can no longer turn over in bed." （原文：黄疸，胆红素500+，肝部多发脓肿，引流引不出来，已经不能翻身了。） |
| T2 Diagnostic Process and Anticipatory Anxiety | "I don't dare to make plans for anything more than two weeks away. Anything further than that, I don't even dare think about it." （原文：不敢安排半个月以后的事情，最多只能管今天明天后天，再往后的事情甚至都不敢想。） "I'll message you again tomorrow, dear. The PET-CT results come back tomorrow, and the pathology still isn't confirmed." （原文：明天再回复你一次亲爱的，petCT结果明天拿，病理目前还没确诊。） |
| T3 Financial Burden and Access to Treatment | "In and out again and again, all kinds of medications plus nutrition support. Even after reimbursement, out-of-pocket costs have already reached hundreds of thousands of yuan." （原文：前前后后进进出出的，各种药加营养，报销自费也花了几十万。） "Money can't save you either. The body can't tolerate any more treatment. It's just reached the end of the road." （原文：有钱也救不了，身体啥治疗都承受不起，就是走到尽头了。） |
| T4 Cancer Treatment Trajectory and Therapeutic Strategies | "Pancreatic cancer was found in August. After three months of chemotherapy combined with immunotherapy, the tail mass and liver lesion shrank by 70 to 80%. CA19-9 started above 12,000; last month's test brought it down to 483." （原文：8月份查出胰腺癌，化疗结合免疫治疗3个月胰尾肿块和肝区肿块缩小70%-80%，ca199起初大于12000，上月检查降到483。） |
| T5 Sleep Disturbance with Day-Night Variation | "No matter day or night, I just can't sleep. It's so painful. Even sleeping pills can't match a normal person's sleep. The cancer cells haven't killed me, but the sleep deprivation nearly has." （原文：不管白天还是黑夜都睡不着，好痛苦，吃了安眠药也抵不上普通人的睡眠，没被癌细胞搞死，但快被睡眠折磨死。） |
| T6 Psychological Distress: Anxiety and Depression | "26 years old, late stage. I never thought I'd be this afraid of dying until this moment actually arrived." （原文：26岁，晚期，这一刻没来临的时候我也没想到自己那么怕死。） "We shuttled through many hospitals and tried many treatments. I regret it more and more; it only added more suffering." （原文：折腾了很多医院，试了很多治疗，越来越觉得后悔，徒增了很多痛苦。） |
| T7 Opioid Use and Pain Management | "The whole body hurts. The oral medication is sustained-release morphine, five tablets now and still no effect." （原文：浑身疼，口服药是盐酸吗啡缓释片，现在吃五片没有效果。） "Only a few days left to live, who cares about addiction? Even if it causes addiction, if it means going peacefully, it's worth it." （原文：都活不了几天了，还在乎成瘾不成瘾，即使成瘾，能走的安详一点也值得。） |
| T8 Healthcare Interaction and Treatment Experience | "It's already late stage. Even in the hospital it's just injections and pills; surgery is no longer possible." （原文：晚期了就算再医院也是打针吃药做不了手术。） "The patient's will to live is very strong, but the doctors are all advising palliative care. It's so hard." （原文：病人求生意志很强，但是医生都劝我临终关怀……好难过。） |
| T9 Death-related Cognition and Existential Distress | "Honestly, right now I don't know whether it's better to be alive or to be gone. It's so tormenting." （原文：说实在，我现在真不知道是活着好，还是离去好，很折磨。） "Cherish the present moment, this is not an empty phrase. Since being diagnosed with late-stage cancer, I have never felt time passing so fast." （原文：珍惜当下时光，这不是句空话。从确诊癌晚期后，从来没有像这样，感受到时间的流速。） |
| T10 Appetite Loss, Nutrition, and Physical Decline | "A quarter of a small bowl of noodles in the morning, half a bowl of soup at noon, two or three bites of rice in the evening. How long can the body hold on like this?" （原文：早上一小碗的四分之一面条，中午半碗汤，晚上两三口米饭，这样的状态还能支撑多久。） |
| T11 Metastasis and Physical Manifestations | "Gastroscopy in April showed no problems. In June, recurrence with peritoneal metastasis, the ascites swelled up like a full-term pregnancy." （原文：4月胃镜无问题。6月复发腹膜转移，腹水涨的跟十月怀胎一样。） "I went hiking and came back to find the lymph node on my neck had swollen to the size of a ping-pong ball, and ended up being told it was late-stage lung cancer." （原文：爬了个山回来，脖子上的结节肿的有乒乓球大，最后说是肺癌晚期，淋巴转移了，骨头上也转移了。） |
| T12 Dyspnea and Respiratory Support | "The suffocation is unbearable. Is death the only way to be free? The oxygen therapy isn't working." （原文：憋的难受，只有死了才能解脱吗？吸氧吸不了。） "Can't lie down to sleep, can only sit up. As soon as I lie down, I have difficulty breathing. Walking two steps and I'm already gasping." （原文：不能躺下睡觉，只能坐着，一躺下就说呼吸困难，走两步路就喘气。） |

* Quotes are presented in English translation with Chinese original in parentheses.
