# Supplementary material for: Characterizing Cancer Symptom Experiences and Related Psychosocial Concerns Through Chinese Social Media Narratives: Computational Text Analysis
Source: J Med Internet Res. 2026 Oct 20;28:e102862. doi: 10.2196/102862 (PMC13683996; doi:10.2196/102862)
Supplement: Supplementary file 7 [file jmir-v28-e102862-s007.docx]

**Multimedia Appendix 7. Topic–Symptom Co-occurrence Matrix: Prevalence of Symptom Dimensions Across the 13 Topics.**

| **Topic** | **n** | **Posts(%)** | **Pain** | **Physical Function Impairment** | **Anxiety** | **Depression** | **Sleep Disturbance** | **Low Energy or Fatigue** |
| --- | --- | --- | --- | --- | --- | --- | --- | --- |
| T0: Family Involvement & Care Decision-Making | 510 | 2.6% | 59.8% | 56.1% | 56.3% | 51.2% | 35.9% | 46.9% |
| T1: Liver Metastasis & Hepatic Dysfunction | 254 | 1.3% | 5.5% | 9.8% | 6.3% | 5.9% | 0.0% | 3.5% |
| T2: Diagnostic Process & Anticipatory Anxiety | 165 | 0.9% | 9.1% | 5.5% | 18.8% | 15.2% | 2.4% | 2.4% |
| T3: Financial Burden & Access to Treatment | 160 | 0.8% | 5.6% | 4.4% | 2.5% | 10.0% | 1.2% | 1.2% |
| T4: Cancer Treatment Trajectory & Therapeutic Strategies | 7200 | 37.1% | 17.3% | 15.5% | 11.3% | 12.5% | 5.1% | 6.6% |
| T5: Sleep Disturbance with Day–Night Variation | 853 | 4.4% | 30.1% | 23.6% | 15.6% | 13.4% | 69.3% | 17.6% |
| T6: Psychological Distress: Anxiety & Depression | 2097 | 10.8% | 20.9% | 13.9% | 29.5% | 34.8% | 6.2% | 7.0% |
| T7: Opioid Use & Pain Management | 360 | 1.9% | 62.2% | 11.7% | 1.4% | 5.0% | 6.9% | 5.0% |
| T8: Healthcare Interaction & Treatment Experience | 4104 | 21.2% | 23.5% | 10.8% | 8.2% | 9.4% | 1.8% | 3.3% |
| T9: Death-related Cognition & Existential Distress | 1344 | 6.9% | 23.0% | 13.9% | 12.5% | 37.5% | 3.4% | 6.7% |
| T10: Appetite Loss, Nutrition & Physical Decline | 1364 | 7.0% | 38.9% | 43.0% | 6.3% | 11.1% | 14.3% | 23.8% |
| T11: Metastasis & Physical Manifestations | 497 | 2.6% | 28.0% | 19.5% | 6.2% | 6.8% | 1.4% | 6.4% |
| T12: Dyspnea & Respiratory Support | 484 | 2.5% | 26.9% | 31.0% | 8.9% | 4.5% | 9.5% | 8.1% |

* Values represent the percentage of posts within each topic in which the corresponding symptom was identified. Posts may be assigned to multiple symptom dimensions. Topics (T0–T12) correspond to the original 13-topic BERTopic solution (see Multimedia Appendix 7 for robustness evidence).
